# Applying a Tripodal Hexaurea Receptor for Binding to an Antitumor Drug, Combretastatin-A4 Phosphate

**DOI:** 10.3390/ma17112570

**Published:** 2024-05-27

**Authors:** Yu Kong, Rong Zhang, Boyang Li, Wei Zhao, Ji Wang, Xiao-Wen Sun, Huihui Lv, Rui Liu, Juan Tang, Biao Wu

**Affiliations:** 1 Key Laboratory of Medicinal Molecule Science and Pharmaceutics Engineering, Ministry of Industry and Information Technology, School of Chemistry and Chemical Engineering, Beijing Institute of Technology, Beijing 102488, China; 3120211210@bit.edu.cn (Y.K.); l18810590212@163.com (R.Z.); zhaochem@bit.edu.cn (W.Z.); 15047166148@163.com (J.W.); 3120215614@bit.edu.cn (X.-W.S.); 3120235794@bit.edu.cn (H.L.); 3220211424@bit.edu.cn (R.L.); 2 College of Chemistry & Pharmacy, Northwest A&F University, Yangling 712100, China; liboyang@nwafu.edu.cn

**Keywords:** anion coordination, tripodal hexaurea receptors, transmembrane transport, phosphate drug

## Abstract

Phosphates play a crucial role in drug design, but their negative charge and high polarity make the transmembrane transport of phosphate species challenging. This leads to poor bioavailability of phosphate drugs. Combretastatin-A4 phosphate (CA4P) is such an anticancer monoester phosphate compound, but its absorption and clinical applicability are greatly limited. Therefore, developing carrier systems to effectively deliver phosphate drugs like CA4P is essential. Anion receptors have been found to facilitate the transmembrane transport of anions through hydrogen bonding. In this study, we developed a tripodal hexaurea anion receptor (**L^1^**) capable of binding anionic CA4P through hydrogen bonding, with a binding constant larger than 10^4^ M^−1^ in a DMSO/water mixed solvent. **L^1^** demonstrated superior binding ability compared to other common anions, and exhibited negligible cell cytotoxicity, making it a promising candidate for future use as a carrier for drug delivery.

## 1. Introduction

Phosphate ions are essential inorganic anions within living organisms. They are the main components of teeth and bones, participating in various bio-chemical activities within the organism, including energy metabolism, and the synthesis of nucleic acids and phospholipids [1]. Therefore, phosphate groups are often added to drug molecules to facilitate specific interactions with cellular targets that involve phosphate-related biochemical activities. They are widely found in various types of drugs, including antiviral, antibacterial, anticancer, and nucleic acid drugs [2,3]. However, under physiological conditions, phosphate groups carry negative charges as anions and are highly hydrophilic. These properties make it difficult for them to be transported across the cell membrane, which is lipophilic and has a negative inside potential [3,4,5]. Therefore, the absorption and bioavailability of phosphate-based drugs are greatly limited [6,7] and their therapeutic efficacy is diminished [8]. Hence, developing delivery tools capable of effectively transporting phosphate-based drugs into cells is of paramount importance.

Combretastatin-A4 phosphate (CA4P) is a monoester phosphate compound exhibiting potent vascular-disrupting antitumor activity. The anticancer potential of CA4P alone or in combination with other antitumor drugs has been tested on a wide variety of tumor models in preclinical and clinical trials [9,10,11]. In addition to the pharmacophore, which includes a trimethoxy ‘A’-ring, a ‘B’-ring comprising a methoxy substituent at C4′, and a cis-ethene bridge amongst the two rings, CA4P also features a disodium phosphate monoester at C3′ in the ‘B’-ring to enhance its water solubility [10]. CA4P exhibits a rapid clearance rate within the body and poor oral absorption, necessitating frequent administration and high drug doses for tumor treatment. However, excess medication concentrations can induce toxicity and adverse reactions. Consequently, the phase III clinical trials of CA4P (NCT02641639 and NCT00507429) were terminated due to severe adverse events, including cardiovascular issues, hematologic toxicity, and tumor pain [10]. These side effects could be bypassed by developing a CA4P delivery strategy to increase cellular uptake, and thus bioavailability. Nanomaterials that are effective in shielding the hydrophilicity and electronegativity of phosphate groups have been employed to improve the bioavailability of CA4P by many groups [12,13,14,15,16,17]. They showed varying degrees of in vivo improvement in antitumor activity and biosafety compared to those of free CA4P. Nonetheless, nanomaterials might exhibit poor biocompatibility, high immunogenicity, and notable significant side effects, limiting their clinical application [18,19]. There is still an urgent need for the development of new chemical delivery tools for the CA4P drug.

Anion receptors are a class of compounds that can bind to anions through non-covalent interactions (primarily through hydrogen bonding) [20,21,22]. The assembly of anions and anion receptors based on anion coordination chemistry have found wide applications in areas such as molecular recognition, catalysis, drug delivery, information storage, and energy materials [20,21,23,24,25]. Coordination with anion receptors could increase the lipophilicity and delocalization of the negative charge of anions. These improvements for anion drugs would facilitate their transmembrane transport, and thus improve their oral absorption [26,27]. Hence, developing anion receptors capable of complexing the anionic drug, CA4P, is important for its clinical translation.

Tripodal hexaurea ligands, derived from the tri(2-aminoethyl)amine (TREN) backbone, exhibit a preorganized structure possessing *C*_3_ symmetry featuring multiple hydrogen bond donors and acceptors. This configuration enables effective topological matching and strong hydrogen bonding with anions with similar symmetry [28]. Therefore, they have been commonly employed for the recognition and extraction of tetrahedral anions, such as sulfate and phosphate [27,29,30,31,32,33,34]. More recently, selective binding to a monoester phosphate, choline phosphate, was achieved by two tripodal hexaurea receptors with aromatic substituents [35].

**Figure 1:**
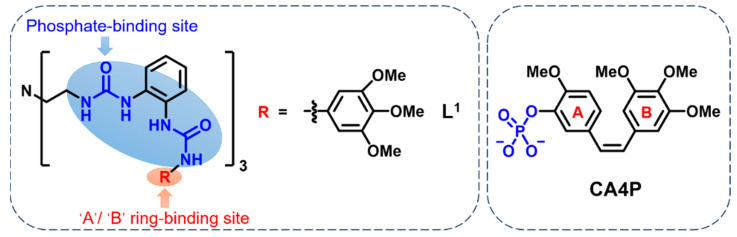
Chemical structures of anion receptor **L^1^** and CA4P.

Inspired by the aforementioned research, one tripodal hexaurea ligand, **L^1^** (Figure 1), was designed to bind to monoester CA4P for the following reasons. (1) The TREN-based hexaurea was chosen to facilitate binding to the phosphate and methoxyl groups of CA4P through hydrogen bonds. (2) A phenyl terminal was incorporated to potentially engage in intermolecular π-π stacking interactions with the ‘A’-ring or ‘B’-ring. Herein, we report the synthesis of the anion receptor, **L^1^**, its CA4P-binding properties, its CA4P-binding selectivity over other common anions, and the cellular cytotoxicity of the anion ligand in vitro.

## 2. Materials and Methods

### 2.1. Reagents and Instruments

All starting chemical materials and solvents were obtained from commercial sources (InnoChem, Beijing, China, Macklin, Shanghai, China, Aladdin, Shanghai, China, etc.), which were used without further purification. Nuclear magnetic resonance spectroscopy (NMR) was tested with a Brucker Advance 400 MHz NMR spectrometer (Karlsruhe, Germany), a BIOBASE CO_2_ incubator QP-80II was used for cell culture, and a Thermo Feld Multiskan FC enzyme marker was used for cytotoxicity testing. DMEM (Dulbecco’s modified Eagle’s Medium), FBS (fetal bovine serum) and penicillin–streptomycin was purchased from Adamas, Shanghai, China. HeLa cells were purchased from Beyotime, Shanghai, China.

### 2.2. Experimental Methods

#### 2.2.1. Chemistry

**Scheme 1:**
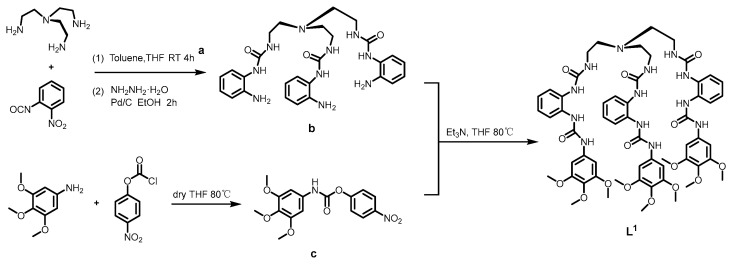
Synthetic scheme for preparing tripodal hexaurea receptor **L^1^**.

Ligand synthesis method (as shown in Scheme 1): *o*-nitrobenzene isocyanate reacted with tris(2-aminoethyl) amine to yield product **a**, which was reduced by hydrazine hydrate to yield product **b**; 3,4,5-trimethoxyaniline reacted with *p*-nitrophenyl chloroformate to obtain product **c**. Compound **b** and compound **c** were reacted at 80 °C and stirred overnight. After about 48 h of reaction, TLC monitored the product **L^1^** (R_f_ = 0.4, CH_2_Cl_2_: CH_3_OH = 15:1), and by-products were generated near the product point. The reaction solution was black-brown, gelatinous and concentrated the reaction solution. Methanol was added to prepare a supersaturated solution, which was heated and stirred for 1 h, then put into the refrigerator, cooled and crystallized, and the filter residue obtained after filtration was pure product **L^1^**. Yield: 48%. Detailed analysis results and spectra are shown in Supplementary Materials.

^1^H NMR nuclear magnetic titration method: The DMSO-*d*_6_ ligand solution was added to a 5 mm test tube at a room temperature of 298 K. An solution of CA4P or anions (50 mM) was then gradually added in equal amounts to the sample solution in the test tube. The sample solution was shaken at room temperature, and the NMR spectra were recorded at room temperature with a Bruker AVANCE II 400M nuclear magnetic instrument. Detailed analysis results and spectra are shown in Supplementary Materials.

Single crystal growth and analytical method: TMA_3_PO_4_ was added to a suspension of ligand (10 mg) in acetonitrile (2 mL). After stirring overnight at room temperature, the mixture was centrifugated and divided into two parts. Next, the mixture was filtered. The obtained clear solution was used to grow crystals. A slow vapor of diethyl ether into the above-mentioned solution provided crystal **L^1^·PO_4_^3−^** within two months. X-ray diffraction data were collected using a Bruker D8 Venture Photon II diffractometer at 180 K with graphite-monochromated Mo Kα radiation (λ = 0.71073 Å). An empirical absorption correction using SADABS was applied for all data (G. M. Sheldrick, Program SADABS: Area-Detector Absorption Correction, 1996, University of Göttingen, Gottingen, Germany). Structures were solved by dual methods using the SHELXS program [36]. All non-hydrogen atoms were refined anisotropically by full-matrix least-squares on F^2^ using the program SHELXL, and hydrogen atoms were included in idealized positions with thermal parameters equivalent to 1.2 times those of the atom to which they were attached. It was noted that three countercations TMA^+^ were confirmed, making the total positive charges +3. Therefore, the charges (negative and positive) of the entire crystal structure were balanced.

#### 2.2.2. Biology

Cell experimental conditions:

(1) Cell culture: HeLa cells were incubated in complete medium (DMEM, supplemented with 10% FBS and 1% penicillin–streptomycin) at 37 °C in an atmosphere containing 5% CO_2_.

(2) Toxicity test method: HeLa cells were seeded in flat-bottomed 96-well plates, 1 × 10^4^ cells per well, with 200 μL complete culture media for 24 h. After removing the old medium, HeLa cells were incubated with different concentrations of complex. All stock solutions were prepared in DMSO (5 mM) and diluted with complete medium. After culturing for 48 h, the cells were washed with PBS (pH = 7.4) two times. A 10 μL Cell Counting Kit-8 (CCK-8) solution and 90 μL DMEM were simultaneously added per well. After 40 min, the absorbance at 450 nm was read by a 96-well plate reader.

## 3. Results and Discussion

### 3.1. Synthesis and Characterization

**L^1^** was obtained with a 48% isolated total yield by reacting the 4-nitrophenoxycarbonyl-modified 3,4,5-trimethoxyphenyl with an aniline-based tripodal triurea precursor. The structure was characterized and confirmed by ^1^H NMR spectroscopy, high-resolution mass spectrometry, and crystal structure. Detailed synthetic procedures and characterization data are included in the Supplementary Materials along with the associated spectra.

### 3.2. Binding Ability of the Anion Recepor to the Phosphate Drug CA4P

**Figure 2:**
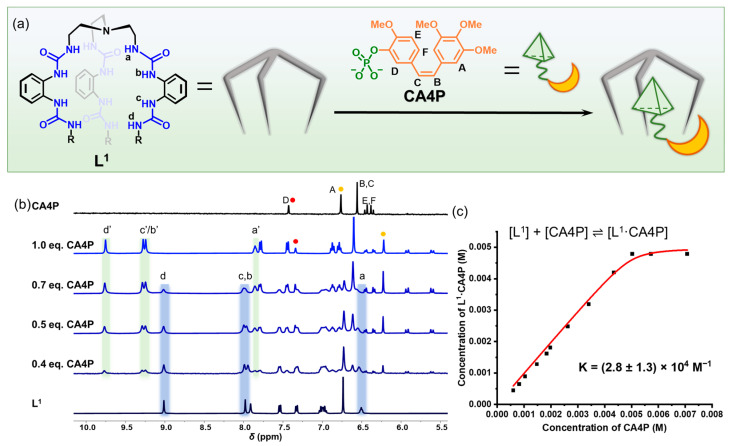
(**a**) Ligand and drug binding process; ^1^H NMR spectra of (**b**) **L^1^** (5 mM) in the presence of various equivalents of CA4P (added as sodium salt) in a mixed solvent of DMSO-*d*_6_/H_2_O (90%/10%, *v*/*v*); a plot of the concentration of the complex of (**c**) **L^1^** as the functions of the concentration of CA4P added, and the corresponding fit of data as a one-to-one binding model.

The binding ability of receptor **L^1^** to the monophosphate drug CA4P was investigated by ^1^H NMR and high-resolution mass spectrometry (Figure 2 and Figure S14). The change in ^1^H NMR spectra of **L^1^** during the titration of CA4P in a mixed solvent of DMSO-*d*_6_/H_2_O (90%/10%, *v*/*v*) is shown in Figure 2b. Upon continuous addition of CA4P, the ^1^H NMR signal of ligand **L^1^** gradually disappeared, and a new set of ^1^H NMR peaks emerged, which can be attributed to the peaks of the **L^1^·CA4P**, indicating that the **L^1^·CA4P** formation followed a slow exchange mechanism on the NMR time scale. Compared to the NH signals (NH_a_ (6.51), NH_b_ (7.91), NH_c_ (7.98), and NH_d_ (9.01)) of ligand **L^1^** alone, the NH signals of the complex **L^1^·CA4P** exhibited significant downfield shifts (NH_a’_ (7.85), NH_b’_ (9.24), NH_c’_ (9.27), and NH_d’_ (9.75)), primarily due to the strong hydrogen bond interactions between CA4P and ligand **L^1^**. When CA4P reached 1.0 equiv. of ligand **L^1^**, changes in the NMR signals of ligand **L^1^** were maximized, indicating that host–guest coordination had reached equilibrium, with the ligand **L^1^** bound to CA4P in a 1:1 binding mode. The binding constant of **L^1^·CA4P** was determined to be larger than 10^4^ M^−1^ (Figure 2c), indicating a strong binding between ligand **L^1^** and CA4P. The binding mode and binding constant of ligand **L^1^** to CA4P are reminiscent of those of the tripodal hexaurea with divalent anions in DMSO/H_2_O mixed solvents reported in the literature [33,37,38]. And then we tried to study the kinetics between **L^1^** and CA4P in 1:1 equiv. in 283 K (10 °C). However, due to the fast reaction rate, once we added the CA4P into a solution of **L^1^**, **L^1^** and CA4P formed a stable host–guest complex in DMSO-*d*_6_/10%H_2_O solution, as shown in Figure S12, indicating fast binding kinetics on the NMR timescale.

**Figure 3:**
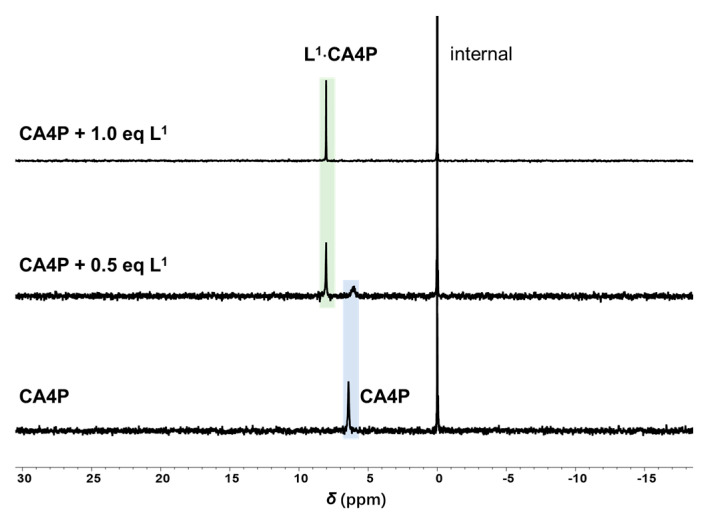
Stacked ^31^P NMR spectra (400 MHz, 298 K, DMSO-*d*_6_/25%H_2_O) of CA4P by adding **L^1^**.

Phosphorus is present in the drug, so we used ^31^P NMR experiments to characterize the binding of **L^1^** and CA4P. Due to the poor solubility of CA4P in DMSO-*d*_6_/10%H_2_O solvent, the ^31^P NMR spectrum of free CA4P alone could not be obtained. Therefore, DMSO-*d*_6_/25%H_2_O solvent was selected for the ^31^P NMR test, and triphenylphosphine was selected as the internal. Results are shown in Figure 3. With the addition of **L^1^**, it was clearly observed that the peak of CA4P (δ = 6.4208 ppm) gradually disappeared and a new peak (δ = 8.0376 ppm) was generated, which we believed to be the peak of the **L^1^·CA4P** complex. When **L^1^** was added to 1.0 equiv., the peak of free CA4P completely disappeared, again indicating that **L^1^** and CA4P combine effectively in a 1:1 ratio.

The stoichiometry of the CA4P complex was further investigated using high-resolution electrospray ionization mass spectrometry in negative ion mode. The peaks observed in the mass spectrum at 1570.5930 (Figure S14 and Table S3) can be assigned to [**L^1^·CA4P** + H^+^]^−^. These results validated the formation of a 1:1 complex, consistent with observations from the ^1^H NMR titration.

**Figure 4:**
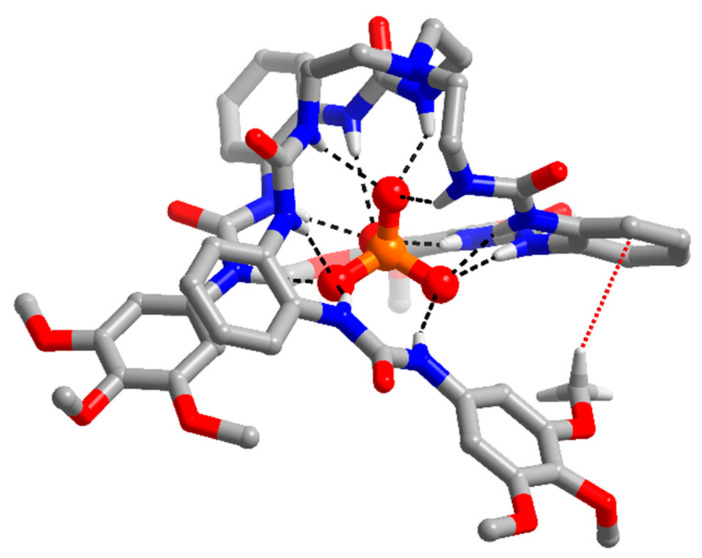
The single crystal structure of the **L^1^·PO_4_^3^**^−^ complex. The black dashed lines and red dashed line indicate hydrogen bonds between phosphates and N–H and a C–H⋯π interaction, respectively.

Although single crystals of the **L^1^·CA4P** complex were not successfully obtained, single crystals of the **L^1^**-phosphate complex, as tetramethylammonium (TMA^+^) salts, suitable for X-Ray crystallography, were obtained through slow vapor diffusion of diethyl ether into a concentrated acetonitrile solution. The crystal structure demonstrated that the tripodal hexaurea ligand bound to the phosphate anion in a 1:1 fashion. In the **L^1^**-phosphate complex, a phosphate ion was encapsulated in the folded cavity, with a distance of 5.02 Å from the top N atom to the central phosphate P atom. The six urea groups were all involved in the coordination to the phosphate anion. Each oxygen atom of the phosphate group formed three hydrogen bonds (N–H⋯O, black dashed line in Figure 4) with three neighboring N–H groups, resulting in a total of twelve hydrogen bonds between the receptor and the phosphate group. Hydrogen bonds (N⋯O distances) ranged from 2.73 to 3.04 Å, with an average distance of 2.83 Å. There was no significant difference in the hydrogen bond distances between the **L^1^**-phosphate complex (2.83 Å) and the reported nitrophenyl modified tripodal hexaurea-phosphate complex (2.80 Å) (CCDC NO. 2290471) [39], indicating a similar phosphate-binding ability of the two tripodal hexaurea. N–H⋯O angles of the **L^1^**-phosphate complex ranged from 136° to 174°, with an average angle of 156°. Similar to C–H⋯π interactions between the terminal 4-nitrophenyl ring and middle phenyl ring in nitrophenyl, modified tripodal hexaurea-phosphate complex, a secondary C–H⋯π interaction (red dashed line in Figure 4) between the C–H of the methyl group at the 3-position in the terminal trimethoxyphenyl ring of **L^1^** and the middle phenyl ring in the neighboring arm, was also observed. Three TMA^+^ cations were displayed in the crystal, making overall +3 charges for charge balance. The crystal structure of the phosphate complex of ligand **L^1^** could offer us the structural information to understand the binding mode of the ligand to the CA4P drug.

**Figure 5:**
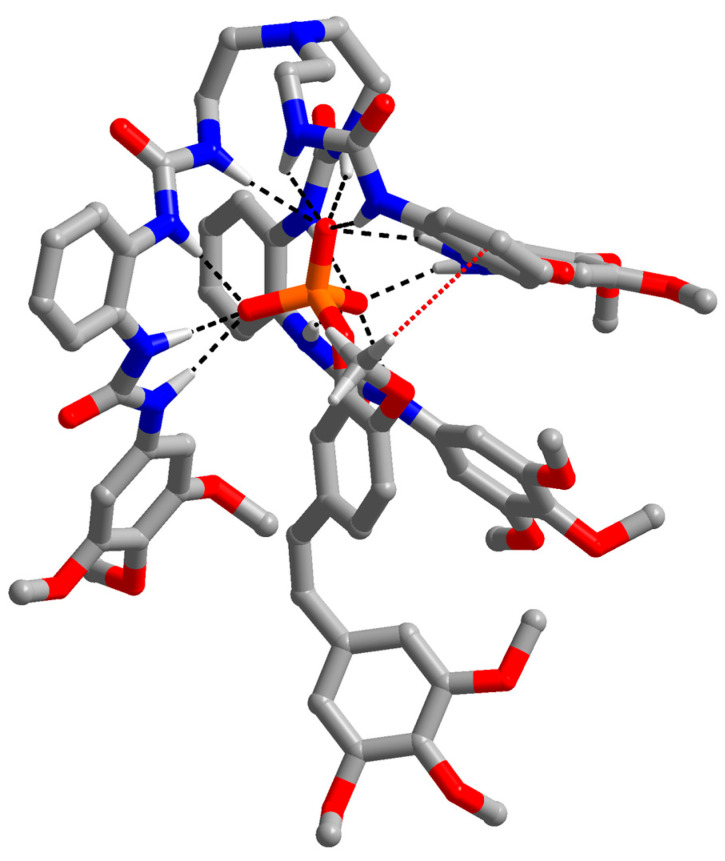
DFT-optimized (B3LYP, 6-31G*) structure of the **L^1^·CA4P** complex. The black and red dashed lines indicate intermolecular hydrogen bonding and the C–H⋯π interaction, respectively.

To gain a deeper insight into the assembly structure between the anion receptor, **L^1^**, and the phosphate drug, CA4P, we performed theoretical calculations by the B3LYP, 6-31G* base group of SPARTAN. The energy-minimized structure of the complex (**L^1^**) with CA4P is shown in Figure 5 and Figure S15. The CA4P guest molecule was engaged in the folded cavity of the ligand mainly through intermolecular hydrogen bonding networks. In the **L^1^·CA4P** complex, there were twelve hydrogen bonds (black dashed line) formed between urea N–H groups and the P–O of CA4P (with N⋯O distances ranging from 2.81 to 3.24, and an average distance of 3.02 Å). Additionally, there was one C–H⋯π interaction between the C–H of the methoxyl ortho to the phosphate group and the middle phenyl ring in the neighboring arm (red dashed line). These interactions collectively resulted in the compact binding of the host to the anionic drug CA4P.

### 3.3. Binding Ability of the Anion Receptor to Other Anions

In practical applications, the presence of various inorganic anions in the human body, such as chloride ions, sulfate ions, and phosphate ions, may compete with CA4P, thereby affecting the binding efficiency of the ligand to CA4P. Therefore, we further investigated the binding constants of ligand **L^1^** to common inorganic anions, including SO_4_^2−^, HPO_4_^2−^, H_2_PO_4_^−^, Cl^−^, and HCO_3_^−^ using ^1^H NMR. To avoid the fast exchange of phosphates among multiple protonation states, we probed the binding ability of inorganic anions in DMSO-*d*_6_ solvent rather than in DMSO-*d*_6_/H_2_O (90%/10%, *v*/*v*) mixed solvents. Results, compiled in Figures S16–S25 and Table 1, showed that the binding constants of **L^1^** to SO_4_^2−^ and HPO_4_^2−^ were both larger than 10^4^ M^−1^. The binding constants of **L^1^** to Cl^−^ and HCO_3_^−^ were about (252 ± 10) M^−1^ and (51 ± 0.4) M^−1^, respectively. However, the binding constant of H_2_PO_4_^−^ could not be obtained due to partial hydrolysis to HPO_4_^2−^ during titration. Notably, the binding constants of **L^1^** to the studied anions in pure DMSO-*d*_6_ were either numerically similar to or smaller than those of **L^1^** to CA4P in DMSO-*d*_6_/H_2_O (90%/10%, *v*/*v*) mixed solvents. Considering the substantial decrease in binding constants observed upon water addition, as documented previously [40], we inferred that the binding affinities of **L^1^** to common anions are notably lower than those of CA4P. This hypothesis was reinforced by the competitive titration in DMSO-*d*_6_. For this investigation, sulfate ions (SO_4_^2−^), which exhibited the highest binding ability, were selected. As illustrated in Figure S25, upon addition of 1.0 equiv. of CA4P to the **L^1^·SO_4_^2−^** complex, ^1^H NMR spectra revealed the disappearance of peaks corresponding to the **L^1^·SO_4_^2−^** complex and the emergence of a new set of peaks corresponding to the **L^1^·CA4P** complex. This observation indicates **L^1^**′s preference for binding to anionic CA4P over other common anions.

### 3.4. Cellular Cytotoxicity of Anion Receptor

**Figure 6:**
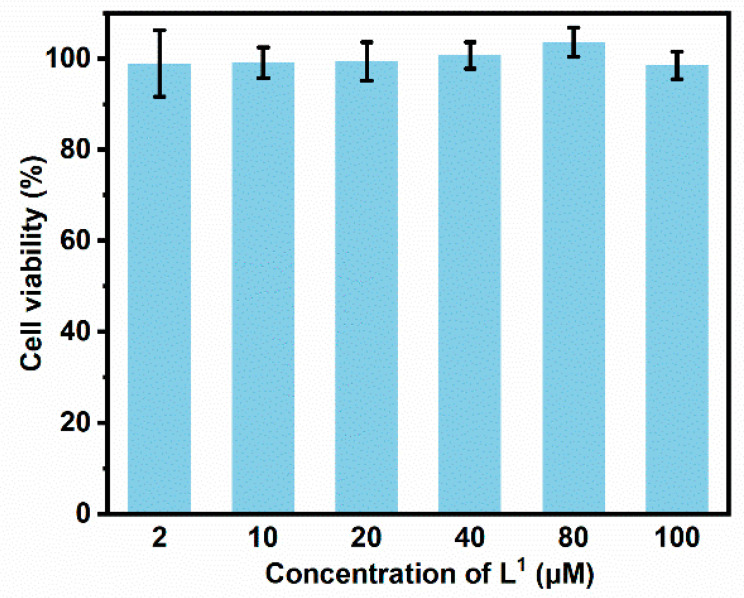
The cytotoxicity of ligand **L^1^** against HeLa cells.

The cytotoxicity of the anion receptor **L^1^** was evaluated against cervical cancer cell line HeLa using the CCK-8 assay, a colorimetric assay for the determination of cell viability in cell proliferation and cytotoxicity experiments [41,42]. Different concentrations of CA4P were co-incubated with HeLa cells for 48 h. As shown in Figure 6, even at an incubation concentration as high as 100 μM, cell viability remained above 90%, indicating that **L^1^** was non-toxic towards HeLa cells. The low cytotoxicity is significant, and established the prerequisite for **L^1^′**s future utilization as a carrier for drug delivery.

## 4. Conclusions

In this work, a tripodal hexaurea ligand **L^1^** was synthesized. Its binding ability to an anionic antitumor drug CA4P via hydrogen bonding was demonstrated through ^1^H NMR, ESI-MS, single-crystal structure analysis, and theoretical calculations. **L^1^** showed superior binding ability over other common anions, such as Cl^−^, HCO_3_^−^, H_2_PO_4_^−^, HPO_4_^2−^, and SO_4_^2−^, and **L^1^** exhibited no cytotoxicity, offering the prerequisite for its future utilization as a carrier for drug delivery. Efforts towards in vitro and in vivo applications of anion receptors for CA4P delivery is ongoing in our laboratories.

## Figures and Tables

**Table 1:** Approximate binding constants (M^−1^) of **L^1^** with anions in DMSO-*d*_6_.

CA4P	SO_4_^2−^	HPO_4_^2−^	H_2_PO_4_^−^	Cl^−^	HCO_3_^−^
^a^ >10^4^	>10^4^	>10^4^	^b^ -	(252 ± 10)	(51 ± 0.4)

^a^ Binding constant of CA4P was measured in DMSO-*d*_6_/H_2_O (90%/10%, *v*/*v*). ^b^ Binding constant could not be fitted.

## Data Availability

The original contributions presented in the study are included in the article/Supplementary Material, further inquiries can be directed to the corresponding authors.

## Supplementary Materials

The following supporting information can be downloaded at: https://www.mdpi.com/article/10.3390/ma17112570/s1, Figure S1. ^1^H NMR spectrum (400 MHz, 298 K, DMSO-*d*_6_) of compound a. Figure S2. ^1^H NMR spectrum (400 MHz, 298 K, DMSO-*d*_6_) of compound b. Figure S3. ^1^H NMR spectrum (400 MHz, 298 K, DMSO-*d*_6_) of compound c. Figure S4. ^1^H NMR spectrum (400 MHz, 298 K, DMSO-*d*_6_) of compound L^1^. Figure S5. ^13^C NMR spectrum (101 MHz, 298 K, DMSO-*d*_6_) of compound a. Figure S6. ^13^C NMR spectrum (101 MHz, 298 K, DMSO-*d*_6_) of compound b. Figure S7. ^13^C NMR spectrum (101 MHz, 298 K, DMSO-*d*_6_) of compound c. Figure S8. ^13^C NMR spectrum (176 MHz, 298 K, DMSO-*d*_6_) of compound L^1^. Figure S9. X-ray structure for the complex of L^1^·PO_4_^3−^ showing overall 1:1 stoichiometry. Secondary C–H⋯π interactions between the C–H of the methyl group at the 3-position in terminal trimethoxyphenyl ring and the middle phenyl ring in the neighboring arm were also observed. Figure S10. Stacked partial ^1^H NMR spectra (400 MHz, 298 K, DMSO-*d*_6_) of receptor L^1^ by adding CA4P as sodium salt. ([L^1^] = 5 mM, [CA4P] = 50 mM) Figure S11. Fitted curve for the ^1^H NMR titration between L^1^ and CA4P, which is derived from Figure S10. It indicates that the binding constant of the ligand to CA4P is larger than 10^4^ M^−1^. Figure S12. Stacked ^1^H NMR spectrum of kinetic study between L^1^ and CA4P in 1:1 equivalent. Figure S13. Stacked ^31^P NMR spectra (400 MHz, 298 K, DMSO-*d*_6_/25%H_2_O) of CA4P by adding ligand L^1^. Figure S14. High-resolution electrospray ionization mass spectrometry in negative ion mode of [L^1^·CA4P + H^+^]^−^. Figure S15. Hydrogen bonding networks seen in DFT-optimized structure of L^1^ for the CA4P binding complex using Spartan 20 at the theory level of B3LYP/6-31G*. Secondary C−H⋯π within phenyl spacer and C−H atoms of terminal phenyl ring are shown on the right. Figure S16. Stacked partial ^1^H NMR spectra (400 MHz, 298 K, DMSO-*d*_6_) of receptor L^1^ by adding chloride as tetrabutylammonium salt (TBACl). ([L^1^] = 2 mM, [TBACl] = 50 mM) Figure S17. Chemical shift changes of proton H_d_, H_c_ and H_b_ during titration. The chloride binding affinity was determined to be (215 ± 7) M^−1^ using Bindfit (v0.5). Figure S18. Stacked partial ^1^H NMR spectra (400 MHz, 298 K, DMSO-*d*_6_) of receptor L^1^ by adding chloride as tetrabutylammonium salt (TBAHCO_3_). ([L^1^] = 2 mM, [TBAHCO_3_] = 50 mM) Figure S19. Chemical shift changes of proton H_d_, H_c_, H_b_ and Ha during titration. The chloride binding affinity was determined to be (51 ± 0.4) M^−1^ using BindFit (v0.5). Figure S20. Stacked partial ^1^H NMR spectra (400 MHz, 298 K, DMSO-*d*_6_) of receptor L^1^ by adding TBA_2_HPO_4_. ([L^1^] = 2 mM, [TBA_2_HPO_4_] = 50 mM) Figure S21. Fitted curve for the ^1^H NMR titration between L^1^ and HPO_4_^2−^, which is derived from Figure S20. It indicates that the binding constant of the ligand to HPO_4_^2−^ is larger than 10^4^ M^−1^. Figure S22. Stacked partial ^1^H NMR spectra (400 MHz, 298 K, DMSO-*d*_6_) of receptor L^1^ by adding TBAH_2_PO_4_. ([L^1^] = 2 mM, [TBAH_2_PO_4_] = 50 mM) Figure S23. Stacked partial ^1^H NMR spectra (400 MHz, 298 K, DMSO-*d*_6_, 2 mM) of chloride binding complex by adding TBA_2_SO_4_ (50 mM). Figure S24. Fitted curve for the competitive titration between sulfate and chloride anions, which is derived from Figure S23. It indicates that sulfate binding affinity is 121-fold stronger than chloride. It can be concluded that the binding constant of ligand and sulfate is larger than 10^4^ M^−1^. Figure S25. Stacked partial ^1^H NMR spectra (400 MHz, 298 K, DMSO-*d*_6_, 2 mM) of sulfate binding complex by adding CA4P (50 mM). Table S1. Crystal data details for obtained structures. Table S2. Hydrogen bonding information in the crystal structure of L^1^·PO_4_^3−^. Table S3. Comparison of the calculated theoretical values and the measured values of the mass for the complexes (acetonitrile/water 10%) Table S4. Approximate binding constants (M^−1^) of L^1^ with anions in DMSO-*d*_6_.
