# Supplementary material for: Applying a Tripodal Hexaurea Receptor for Binding to an Antitumor Drug, Combretastatin-A4 Phosphate
Source: Materials (Basel). 2024 May 27;17(11):2570. doi: 10.3390/ma17112570 (PMC11173554; doi:10.3390/ma17112570)
Supplement: Supplementary file 1 [file materials-17-02570-s001.zip › materials-2994505-supplementary.pdf]

Electronic Supplementary Information (ESI)

Contents

S1. General information....3

S2. Synthetic procedures of hexaurea receptors 3

S3. Single crystal X-ray diffraction structures....8

S4. ^{1}H NMR studies of L^1 binding to CA4P....11

S5. ^{31}P NMR studies of L^1 binding to CA4P 13

S6. Mass studies of anion receptors binding to CA4P....14

S7. Computational studies of anion receptor L^1 binding to CA4P 15

S8. ^{1}H NMR studies of L^1 binding to anions....15

S9. Cell experimental conditions 21

S1. General information

All starting materials and solvents were obtained from commercial sources (Beijing InnoChem, Aladdin, Macklin Science & Technology Co., Ltd.), which were used without further purification. ^{1}H NMR spectra were recorded on Bruker AVENCE AV II-400/700 MHz spectrometer at 298 K. ^{1}H NMR chemical shifts were reported relative to residual solvent peaks (^{1}H NMR: 2.50 ppm for DMSO-d_6, 7.26 ppm for $CDCl_3$). High resolution mass spectrometry data were obtained by AGILENT Q-TOF 6520. Single crystal X-ray data were recorded on Bruker D8 Venture Photon II diffractometer. All aqueous solutions were prepared by using ultrapure water (18.25 MΩ·cm).

The phosphate salts are not commercially available and were prepared by acid-base reaction from corresponding tetraalkylammonium hydroxide solution (water) and phosphoric acid. The phosphoric acid (H_3PO_4) was placed in a small vial and certain amounts of hydroxide was added. The mixture was diluted to 1 mL and the prepared solution was used directly or diluted to a certain concentration for titration.

TMA_3PO_4 (water): H_3PO_4 was mixed with three equivalents of TMAOH (40% wt, H_2O).

TBA_3PO_4 (DMSO): $TBAH_2PO_4$ was mixed with two equivalents of TBAOH (40% wt, H_2O).

TBA_2HPO_4 (DMSO): $TBAH_2PO_4$ was mixed with one equivalent of TBAOH (40% wt, H_2O).

S2. Synthetic procedures of hexaurea receptors

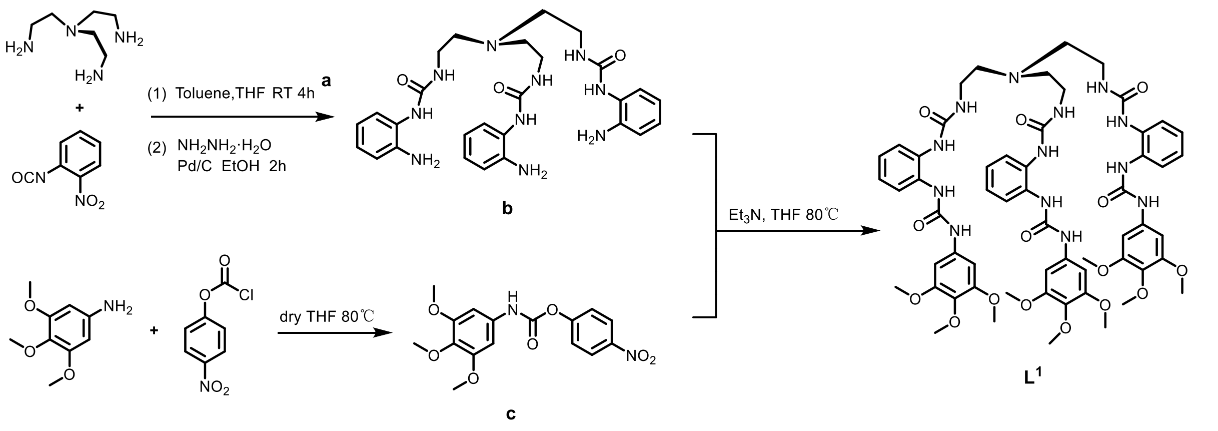

Scheme S1. Synthetic scheme of preparing tripodal hexaurea receptor **L**^{1}.

Synthesis of compound a:

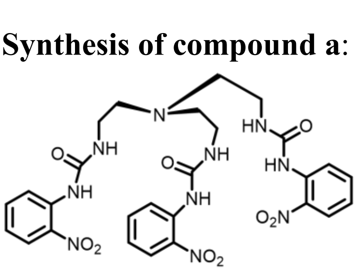

o-nitrobenzene isocyanate (3.0 g, 18.3 mmol, 3.2 equiv.) was placed in a 250 mL round-bottomed flask and dissolved in toluene (70 mL). A solution of tris(2- aminoethyl)amine (tren, 0.84 g, 5.7 mmol, 1 equiv.) in THF (20 mL) was added into the flask by using a dropping funnel (2 seconds per droplet). Obvious precipitation occurred once tren react with isocyanate. The reaction wasstirred at room temperature for 4 hours and monitored by TLC. The precipitates were separated by filtration and washed with toluene (10 mL × 3) and diethyl ether (10 mL × 3). The obtained yellow powder was further dried over vacuum dried and isolated as compound **a** (3.5 g). Yield: 96%. ^{1}H NMR (400 MHz, DMSO-d_6) δ 9.34 (s, 1H), 8.24 (d, J = 8.6 Hz, 1H), 7.99 (dd, J = 8.4, 1.6 Hz, 1H), 7.65 – 7.50 (m, 1H), 7.48 (s, 1H),

7.08 (ddd, J = 8.5, 7.2, 1.3 Hz, 1H), 3.21 (q, J = 6.4 Hz, 2H), 2.63 (t, J = 6.7 Hz, 2H). ^{13}C NMR (101 MHz, DMSO-d_6) δ 154.76, 137.25, 136.28, 135.30, 125.70, 122.54, 121.79, 54.03, 40.61, 40.40, 40.19, 39.98, 39.78, 39.57, 39.36, 38.23.

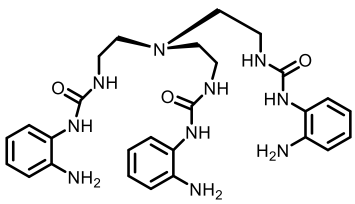

Synthesis of compound b: Compound **a** (3.0 g, 4.7 mmol) was placed in double-necked, round-bottomed flask and suspended in ethanol (500 mL). Pd/C (0.7 g) was added into the flask. The reaction mixture was heated to 90°C and stirred for 20 mins until all the starting material got dissolved. Hydrazine monohydrate (7.5 mL) was added dropwise using dropping funnel. The reaction mixture was further stirred at the same temperature for 2 hours until all the reactant was consumed. Before cooling to room temperature, the Pd/C was removed by filtration over celite. The obtained filtrate was concentrated by evaporation and white powder precipitated. The white powder was subsequently washed by cold ethanol (10 mL) and diethyl ether (10 mL × 3). Compound (10 mL × 3) was obtained as white powder, 1.9 g. Yield, 73%. ^{1}H NMR (400 MHz, DMSO-d_6) δ 7.59 (s, 1H), 7.22 (d, J = 7.9 Hz, 1H), 6.81 – 6.75 (m, 1H), 6.68 (d, J = 9.4 Hz, 1H), 6.53 – 6.48 (m, 1H), 6.19 (t, J = 5.5 Hz, 1H), 4.68 (s, 2H), 3.17 (q, J = 6.4 Hz, 2H), 2.58 (t, J = 6.6 Hz, 2H). ^{13}C NMR (101 MHz, DMSO-d_6) δ 156.53, 141.28, 125.81, 124.48, 124.16, 117.18, 116.22, 54.64, 40.61, 40.40, 40.19, 39.98, 39.77, 39.56, 39.36, 38.28.

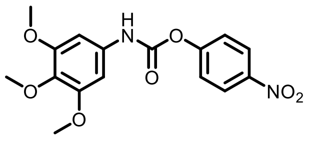

Synthesis of compound c: Under N_2 atmosphere, 3,4,5-trimethoxyaniline (2.0 g, 10.9 mmol, 1 equiv.) was placed in a 250 mL double-necked, round-bottomed flask and suspended in dry THF (70 mL). The reaction was heated to 80°C, and p-nitrophenyl chloroformate (2.62g, 13.0 mmol, 1.2 equiv.) in dry THF (30 mL) was added dropwise over 30 mins. The reaction mixture was stirred at the same temperature for 14 hours. The target product **b** was generated monitored by TLC (R_f = 0.5, CH_2Cl_2: EA = 20:1). The reaction solution is dark green. Then the reaction solution was concentrated to 2 mL, and 50 mL ethyl ether was added. Then it was stored in the refrigerator overnight. The precipitants were filtered and it was the target product c, with a yield of 95%. ^{1}H NMR (400 MHz, DMSO-d_6) δ 10.36 (s, 1H), 8.31 (d, J = 9.1 Hz, 2H), 7.53 (d, J = 9.1 Hz, 2H), 6.88 (s, 2H), 3.74 (s, 6H), 3.63 (s, 3H). ^{13}C NMR (101 MHz, DMSO-d_6) δ 156.00, 153.41, 151.01, 145.04, 134.62, 134.02, 125.69, 123.39, 97.07, 60.59, 56.21, 40.61, 40.40, 40.20, 39.99, 39.78, 39.57, 39.36.

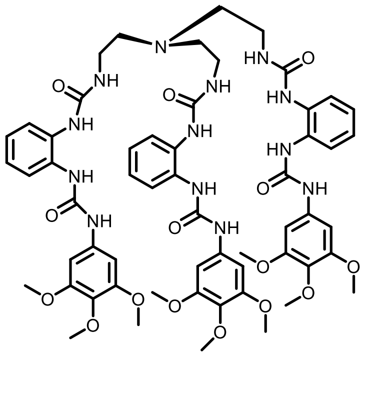

Synthesis of compound L^1: Under N_2 atmosphere, compound **b** (0.3 g, 0.55 mmol, 1 equiv.) and compound **c** (761.4 mg, 2.2 mmol, 4 equiv.) was placed in a 250 mL double-necked, round-bottomed flask and suspend in dry THF (100 mL) and 220 mg Et_3N. The reaction was heated to 80°C and stirred overnight. After about 48 h of reaction, TLC monitored the product **L^1** (R_f = 0.4, CH_2Cl_2: CH_3OH = 15:1), and the by-products were generated near the product point. The reaction solution is black-brown gelatinous and concentrates the reaction solution. Methanol was added to prepare a supersaturated solution, heated and stirred for 1 h, then put into

the refrigerator, cooled and crystallized, and the filter residue obtained after filtration was pure product **L¹**. Yield: 48%. ^{1}H NMR (400 MHz, DMSO-d_6): δ = 9.07 (s, 1H), 7.99 (s, 1H), 7.92 (s, 1H), 7.59 (d, J = 7.7 Hz, 1H), 7.39 (d, J = 9.6 Hz, 1H), 7.05 – 6.95 (m, 2H), 6.79 (s, 2H), 6.50 (t, J = 5.4 Hz, 1H), 3.71 (s, 6H), 3.59 (s, 3H), 3.25 – 3.16 (m, 2H), 2.61 (d, J = 6.8 Hz, 2H). ^{13}C NMR (176 MHz, DMSO-d_6) δ 156.71, 153.55, 153.27, 136.59, 132.73, 131.79, 124.21, 96.27, 60.56, 56.06, 54.44, 40.33, 40.22, 40.10, 39.98, 39.86, 39.74, 39.62. Calculated mass for $[C_{57}H_{69}N_{13}O_{15}+Cl]^-$ at 1210.4730, found: 1210.4735.

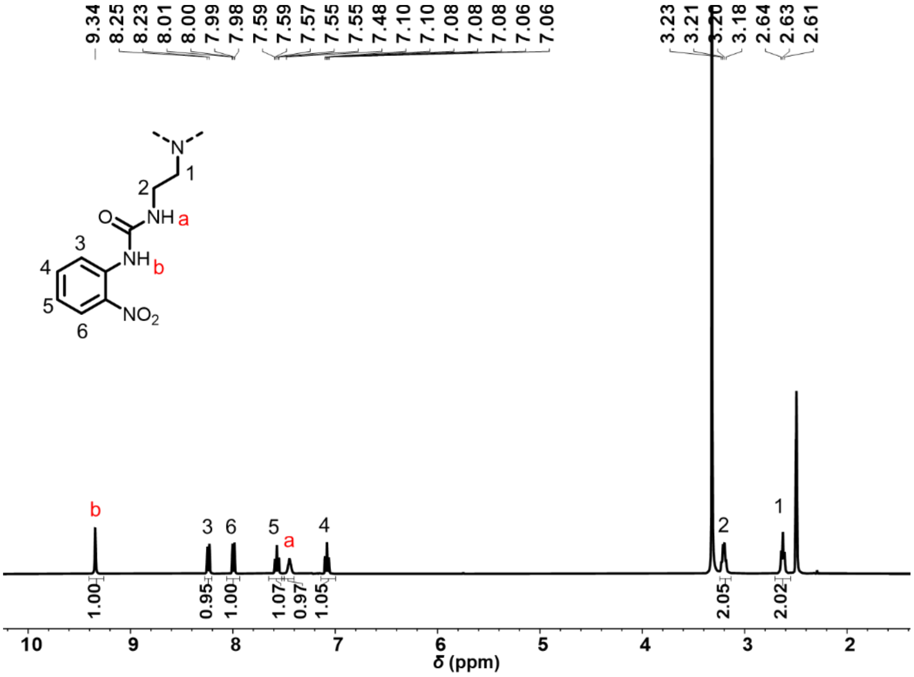

Figure S1. ^{1}H NMR spectrum (400 MHz, 298 K, DMSO-d_6) of compound **a**.

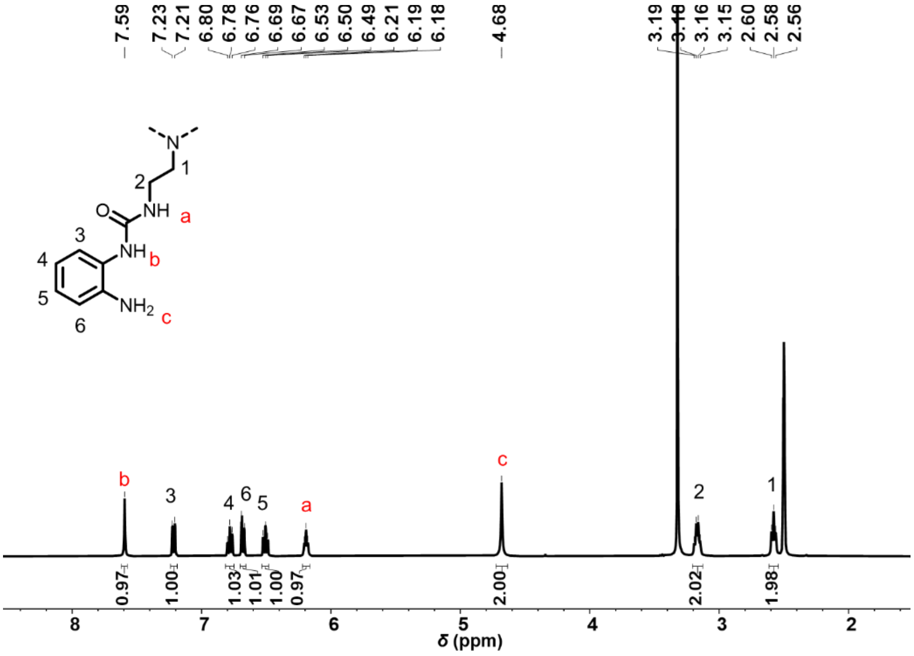

Figure S2. ^{1}H NMR spectrum (400 MHz, 298 K, DMSO-d_6) of compound **b.**

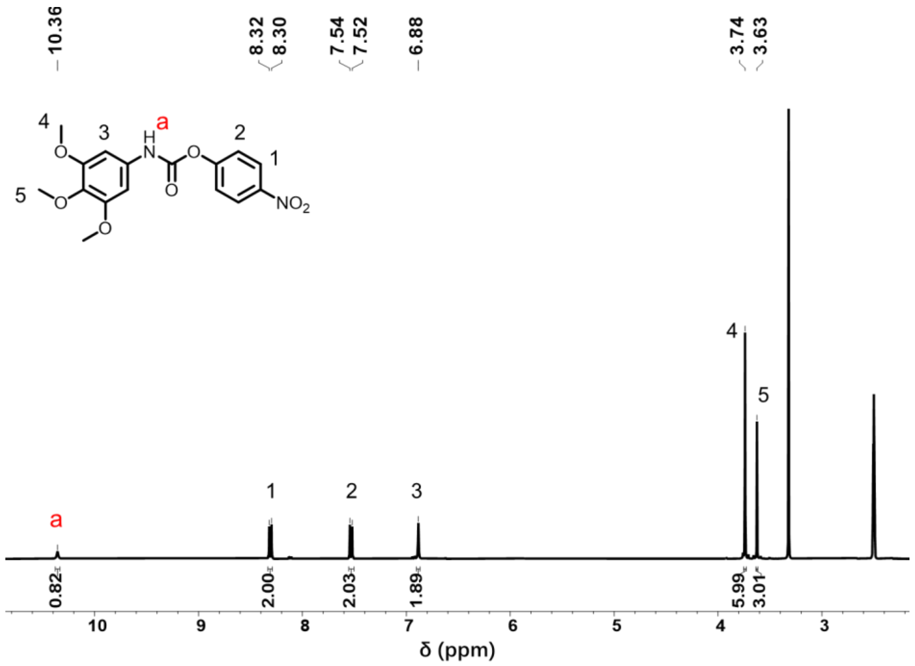

Figure S3. ^{1}H NMR spectrum (400 MHz, 298 K, DMSO-d_6) of compound **c.**

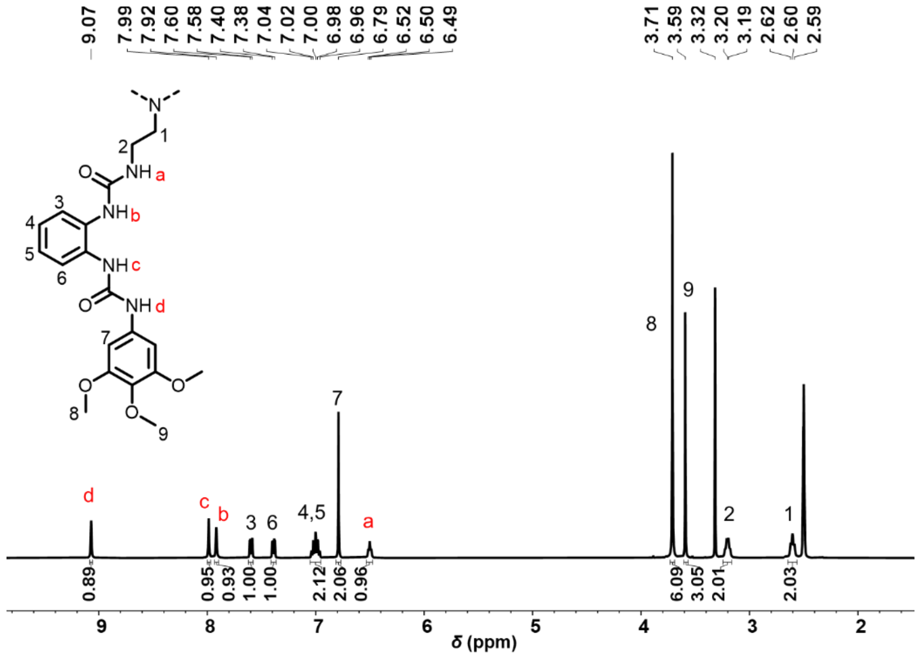

Figure S4. ^{1}H NMR spectrum (400 MHz, 298 K, DMSO-d_6) of compound **L[1]**.

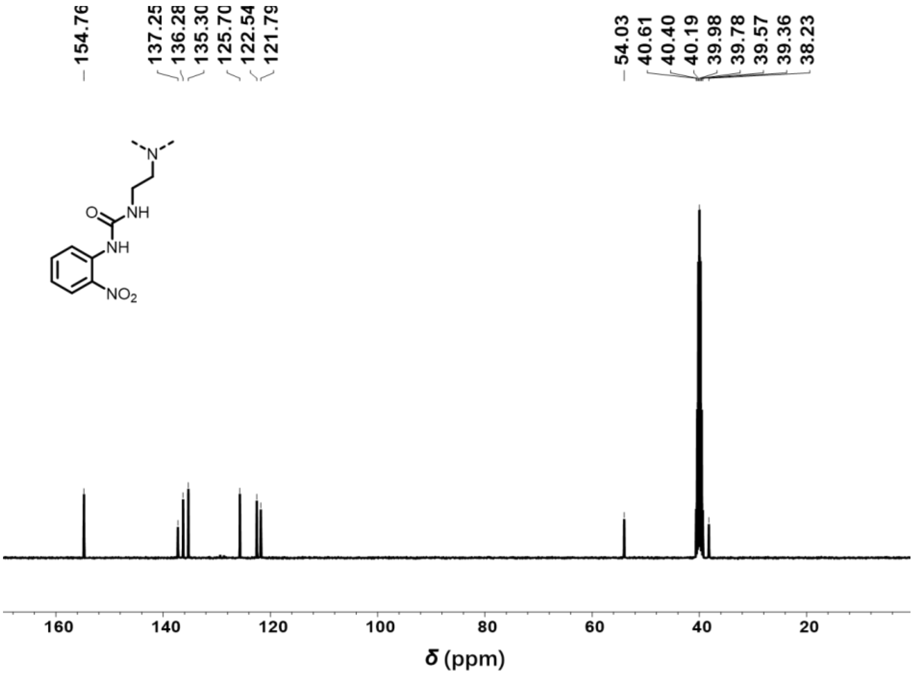

Figure S5. ^{13}C NMR spectrum (101 MHz, 298 K, DMSO-d_6) of compound **a**.

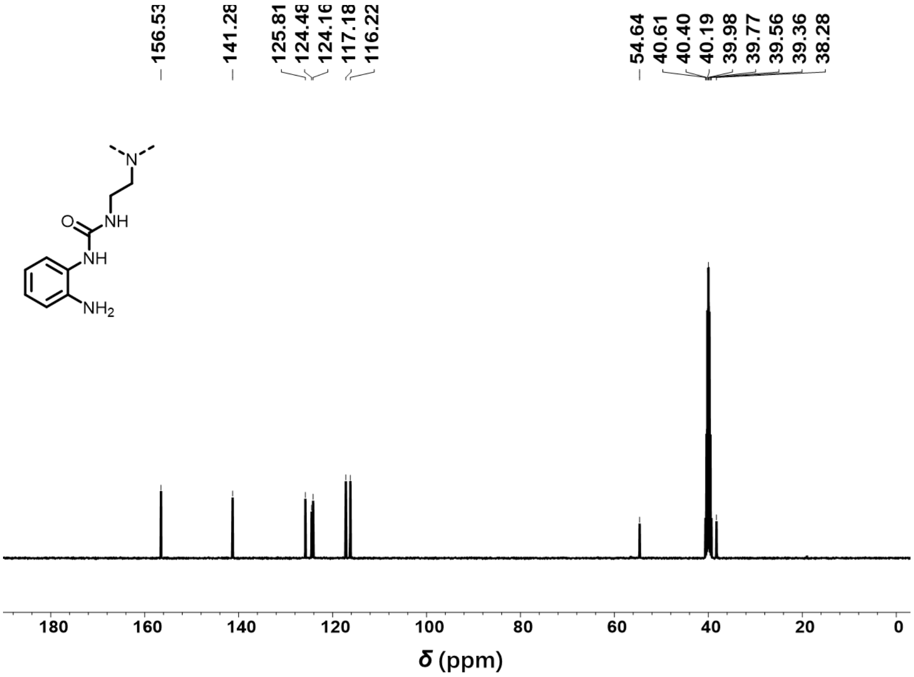

Figure S6. ^{13}C NMR spectrum (101 MHz, 298 K, DMSO-d_6) of compound **b**.

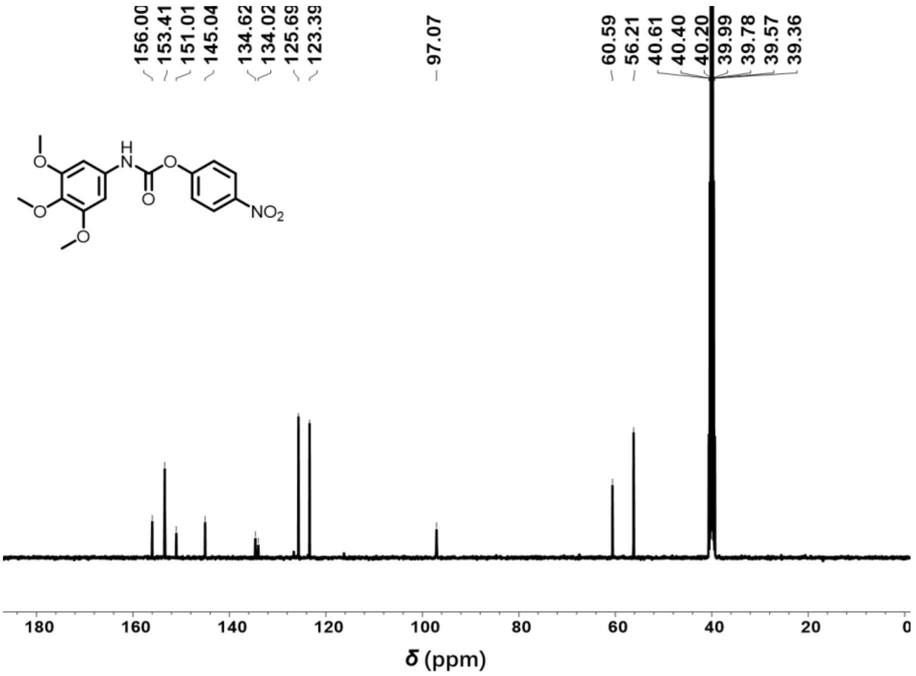

Figure S7. ^{13}C NMR spectrum (101 MHz, 298 K, DMSO-d_6) of compound **c**.

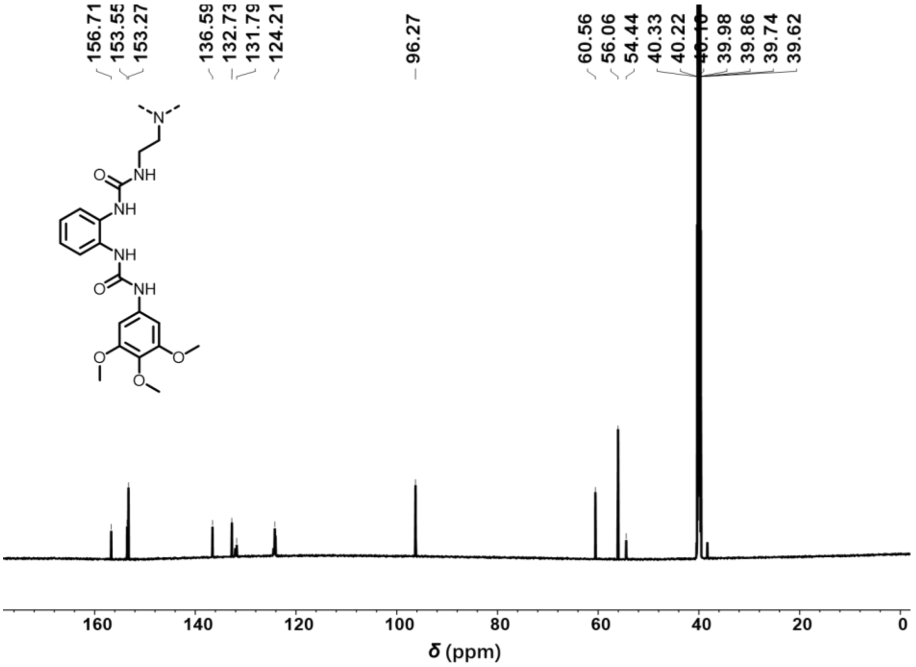

Figure S8. ^{13}C NMR spectrum (176 MHz, 298 K, DMSO-d_6) of compound $\mathbf{L^1}$.

S3. Single crystal X-ray diffraction structures

TMA_3PO_4 was added to a suspension of ligand (10 mg) in acetonitrile (2 mL), then divide the mixture in to two parts after stirring overnight at room temperature in centrifugate. Then the mixture was centrifugate and filtered. The obtainer clear solution was used to crystals growing. Slow vapor of diethyl ether in to above-mentioner solution provided crystal of $L^1\cdot PO_4^{3-}$ within two months. X-ray diffraction data were collected on a Bruker D8 Venture Photon II

diffractometer at 180 K with graphite-monochromated Mo Kα radiation (λ = 0.71073 Å). An empirical absorption correction using SADABS was applied for all data (G. M. Sheldrick, Program SADABS: Area-Detector Absorption Correction, 1996, University of Göttingen, Germany). The structures were solved by the dual methods using the SHELXS program (G. Sheldrick, Acta Cryst. A, 2008, 64, 112-122). All non-hydrogen atoms were refined anisotropically by full-matrix least-squares on F^2 using the program SHELXL, and hydrogen atoms were included in idealized positions with thermal parameters equivalent to 1.2 times those of the atom to which they were attached. It was noted that three countercations TMA^+ were confirmed, making the total positive charges of +3. Therefore, the charges (negative and positive) of entire crystal structure are balanced.
The crystal data (CCDC 2338000) and refinement details are given in Table S1.

Table S1 Crystal data details for obtained structures.

Complex	$L^1 \cdot PO_4^{3-}$
CCDC	2338000
Empirical formula	$C_{152}H_{231}N_{39}O_{41}P_2$
Formula weight	3322.68
Crystal system	monoclinic
Space group	$P2_1/c$
a (Å)	24.513(3)
b (Å)	30.461(4)
c (Å)	23.136(3)
α(deg)	90
β(deg)	92.603(4)
γ(deg)	90
V (Å^3)	17254(4)
Z	4
T (K)	180
F(000)	7096.0
D_{calc},g/cm^3	1.279
Total no. of date	31870
Crystal size (mm)	0.24×0.22×0.18
Completeness to θ	0.986
θ range	4.268 to 51.116
μ /mm^{-1}	0.111
Data/restraints/	31870/102
Parameters	2194

GoF on F^2	1.026
R1	0.0875
wR_2	0.2092

Table S2 Hydrogen bonding information in the crystal structure of $L^1 \cdot PO_4^{3-}$.

D-H···*A*	d(D-H)	d(H···*A*)	d(D···*A*)	∠DH*A*
N2-H2···O16	0.88	2.12	2.844(5)	139
N3-H3···O17	0.88	2.13	2.976(5)	162
N4-H4···O17	0.88	1.91	2.730(5)	154
N5-H5···O19	0.88	2.09	2.935(5)	162
N6-H6···O16	0.88	2.08	2.784(5)	136
N7-H7···O19	0.88	2.02	2.872(4)	162
N8-H8···O19	0.88	1.92	2.790(4)	171
N9-H9···O18	0.88	1.91	2.777(4)	166
N10-H10···O16	0.88	2.03	2.749(5)	138
N11-H11···O18	0.88	2.22	3.042(5)	155
N12-H12···O18	0.88	1.86	2.741(5)	174
N13-H13···O17	0.88	1.95	2.778(5)	156
average	0.88	2.02	2.835(5)	156

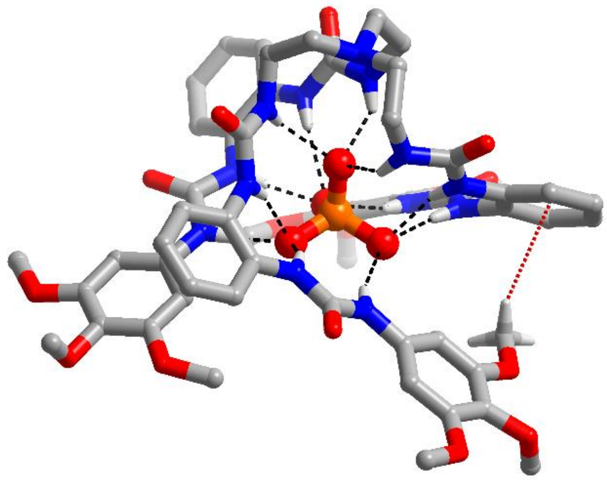

Figure S9. X-ray structure for the complex of $L^1 \cdot PO_4^{3-}$ showing overall 1:1 stoichiometry. Secondary C-H···π interactions between the C-H of the methyl group at the 3-position in terminal trimethoxyphenyl ring and the middle phenyl ring in the neighboring arm were also observed.

S4. ^{1}H NMR studies of L^1 binding to CA4P

To determine the binding ability of the ligand to the drug, nuclear magnetic titration and high-resolution mass spectrometry were used. First, nuclear magnetic titration was used. At room temperature 298 K, prepared DMSO-d_6 ligand solution was added to a 5 mm tube. Solvent system set to DMSO-d_6/H_2O (90%/10%, *v/v*). Then, the aqueous solution of CA4P (50 mM) was gradually added to the sample solution in the tube according to the equivalent number. The sample solution in the magnetic tube was shaken and the ^{1}H NMR spectra were recorded at room temperature using a Bruker AVANCE II 400M nuclear magnetic instrument. The results were shown in Figure S10 and S11, which indicated that CA4P and the ligands were both slowly exchanged. The characteristic peaks with obvious peak position changes and accurate integration were selected for integration, and the corresponding concentration of the complex was calculated according to the integral area. The obtained data were fitted and analyzed using Origin to obtain the binding constants of the drug and the ligand.

The specific calculation process is as follows:

Slow exchange titration reaction equation:

$$L + CA4P \rightleftharpoons L{\cdot}CA4P$$

For the calculation, we defined these:

the total concentration of CA4P added is **x**，initial concentration of L is **b**,

[L·CA4P] = **y**, [CA4P] = **x-y**, [L] = **b-y**

the equilibrium constant *K* can be represented as

$$K = \frac{[L{\cdot}CA4P]}{[L]*[CA4P]} = \frac{y}{(b-y)(x-y)} = \frac{y}{y^2-(x+b)y+bx}$$

$$\frac{1}{K} = \frac{y^2-(x+b)y+bx}{y}$$

$$y^2 - (\frac{1}{K}+x+b)y+bx=0$$

$$y = \frac{(\frac{1}{K}+x+b) \pm \sqrt{(\frac{1}{K^2}+x+b)^2-4bx}}{2}$$

Thus, the equilibrium constant *K* can be determined based on the following equation using Origin.

$$y = \frac{(xK+bK+1) \pm \sqrt{(1+bK+xK)^2-4bx*K^2}}{2K}$$

Through the integration changes of peak **d**, the binding constants of CA4P and L^1 was larger than 10^4 M^{-1}.

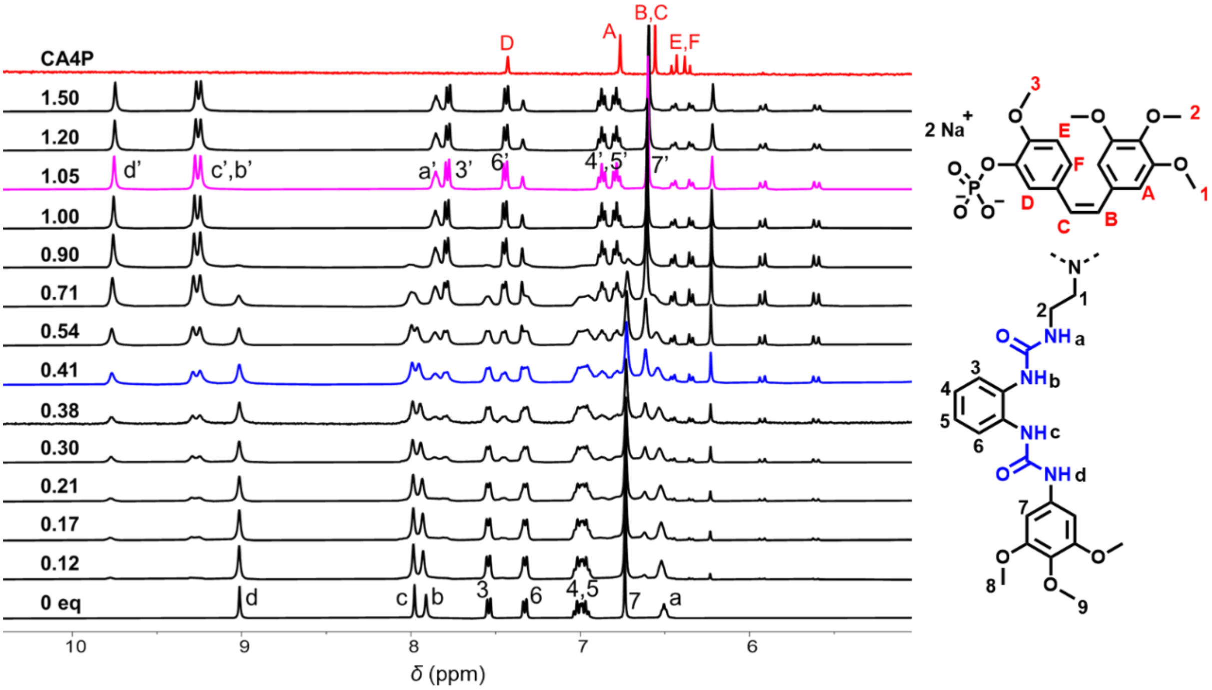

Figure S10. Stacked partial ^{1}H NMR spectra (400 MHz, 298 K, DMSO-d_6) of receptor $\mathbf{L}^1$ by adding CA4P as sodium salt. ([$\mathbf{L}^1$] = 5 mM, [CA4P] = 50 mM)

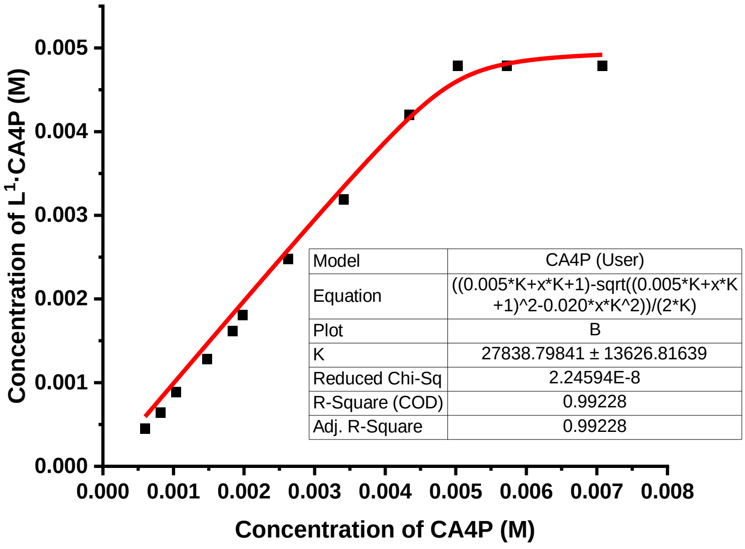

Figure S11. Fitted curve for the ^{1}H NMR titration between L^1 and CA4P, which is derived from Figure S10. It indicates that the binding constant of the ligand to CA4P is larger than 10^4 M^{-1}.

We have tried *via* ^{1}H NMR experiments to study the kinetics between L^1 and CA4P in 1:1 equivalent in 283 K (10ºC). However, due to the fast reaction rate, once we added the CA4P into solution of L^1, L^1 and CA4P forming a stable host-guest complex in DMSO-d_6/10%H_2O solution as shown in Figure S12, indicating the fast binding kinetics on the NMR timescale.

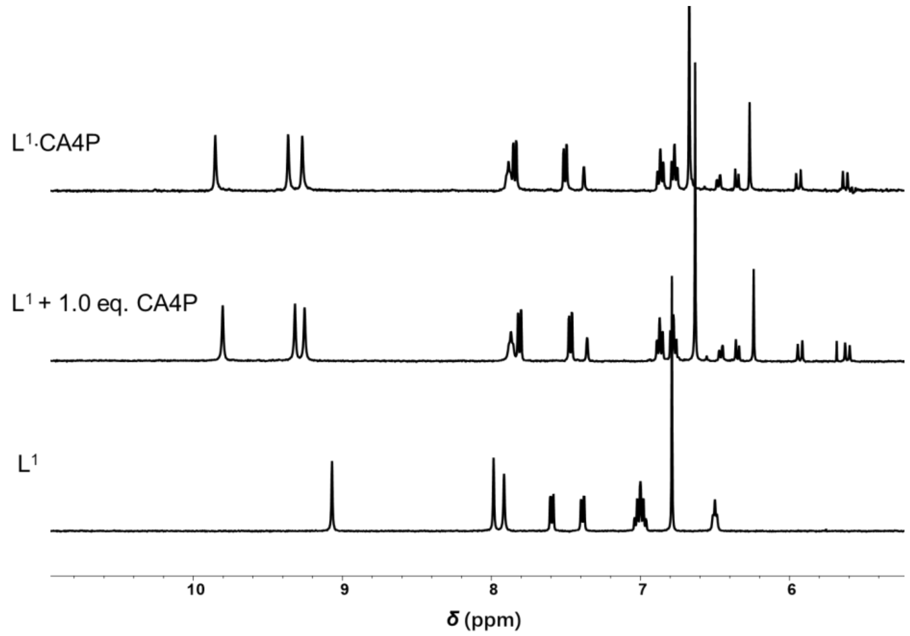

Figure S12. Stacked ^{1}H NMR spectrum of kinetic study between L^1 and CA4P in 1:1 equivalent.

S5. ^{31}P NMR studies of L^1 binding to CA4P

Due to the poor solubility of CA4P in DMSO-d_6/10%H_2O solvent, the ^{31}P NMR spectrum of free CA4P alone could not be obtained. Therefore, DMSO-d_6/25%H_2O solvent was selected for the ^{31}P NMR test, and triphenylphosphine was selected as the internal. At room temperature 298 K, the internal was added, and then the 50 mM CA4P solution prepared in advance was added into the nuclear magnetic tube with a diameter of 5 mm according to the ratio, and the final drug concentration was 5 mM. The ligands are then gradually added to the nuclear magnetic tube (solid) in accordance with the equivalent number and fully shaken. ^{31}P NMR spectra were recorded with Bruker AVANCE II 400M nuclear magnetometer.

The results are shown in the Figure S13. With the addition of L^1, it was clearly observed that the peak of CA4P (δ= 6.4208 ppm) gradually disappeared and a new peak (δ= 8.0376 ppm) was generated, which we believed to be the peak of the L^1·CA4P complex. When L^1 was added to 1 equiv., the peak of free CA4P completely disappeared, again indicating that L^1 and CA4P combine effectively in a 1:1 ratio.

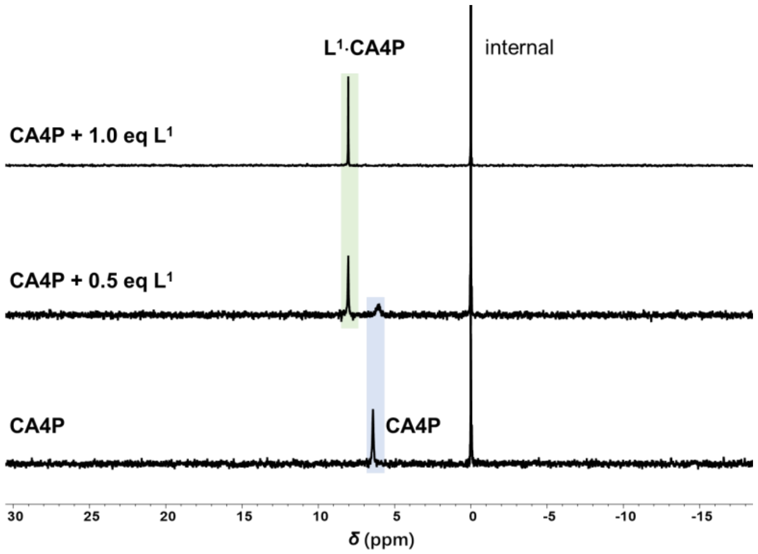

Figure S13. Stacked ^{31}P NMR spectra (400 MHz, 298 K, DMSO-d_6/25%H_2O) of CA4P by adding ligand L^1.

S6. Mass studies of anion receptors binding to CA4P

Experimental methods and conditions of mass spectrometry monitoring: In order to clarify the binding ratio of ligand and CA4P under solution conditions, high-resolution mass spectrometry method was used to verify. Solvent condition: 90%/10% (*v/v*) CH_3CN/H_2O, mass spectrometry mode: Q-TOF, ESI^-. The ligands of a certain mass were weighed and dissolved in acetonitrile solution, into which 1 equivalent CA4P aqueous solution was added and fully stirred to obtain the complex mass spectra as shown in Figure S14. The difference between the m/z peak value and the theoretical value is within the error range, which proves that the ligand and CA4P adopt a 1:1 binding mode, and the values are summarized in Table S3.

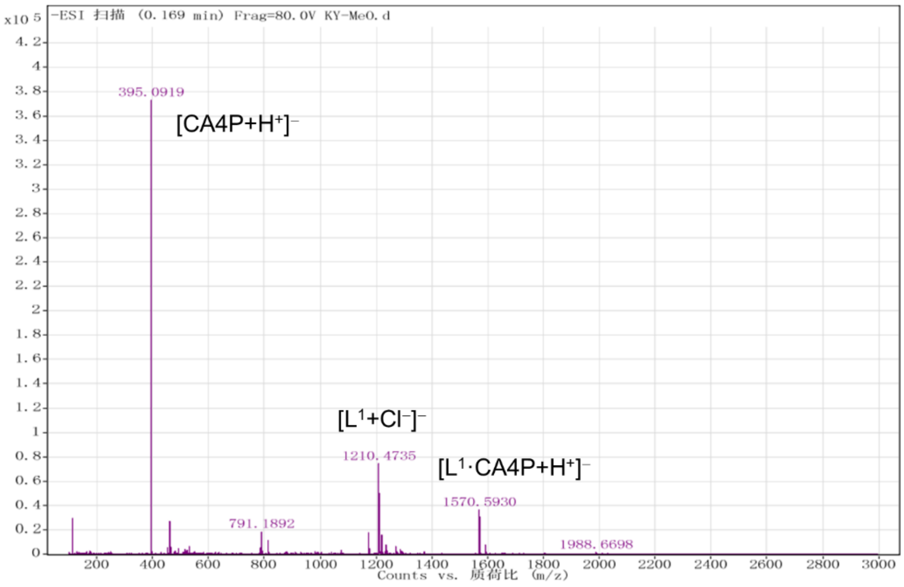

Figure S14. High-resolution electrospray ionization mass spectrometry in negative ion mode of $[L^1{\cdot}CA4P + H^+]^-$.

Table S3 Comparison of the calculated theoretical values and the measured values of the mass for the complexes (acetonitrile/water 10%)

Tested combinations	Theoretical values m/z	Measured values m/z
[**L^1·CA4P** + H$^+$]$^-$	1570.5937	1570.5930

S7. Computational studies of anion receptor L^1 binding to CA4P

To get structural information for the complex upon binding to CA4P, computational calculations were conducted by using Spartan. The structure optimized at the theory level of B3LYP, 6-31G*.

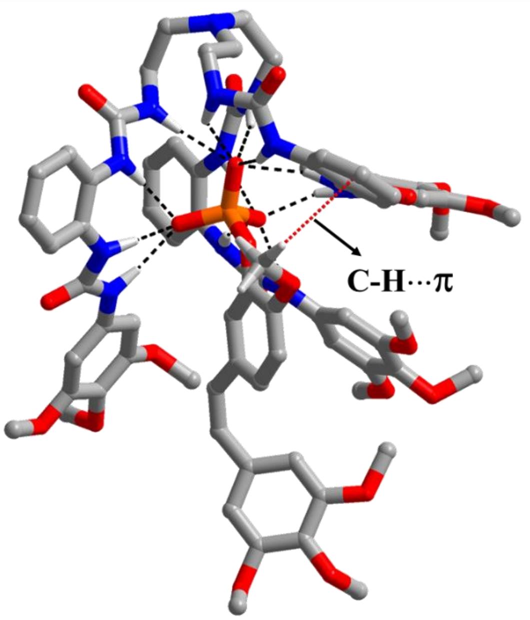

Figure S15. Hydrogen bonding networks seen in DFT-optimized structure of **L^1** for the CA4P binding complex using Spartan 20 at the theory level of B3LYP/6-31G*. Secondary C-H···π within phenyl spacer and C−H atoms of terminal phenyl ring are shown on the right.

S8. ^{1}H NMR studies of L^1 binding to anions

To determine the binding ability of the ligand to the anions, nuclear magnetic titration was used. At room temperature 298 K, 500 μL of the prepared 2 mM DMSO-*d*$_6$ ligand solution was added to a 5 mm tube. Then, the DMSO-*d*$_6$ solution of anions (50 mM) was gradually added to the sample solution in the tube according to the equivalent number. The sample solution in the magnetic tube was shaken and the ^{1}H NMR spectra were recorded at room temperature using a Bruker AVANCE II 400M nuclear magnetic instrument. The results were shown in Figure S16 – S25.

The results in Figure S16 indicated that Cl$^-$ and the ligand were quickly exchanged. The hydrogen peaks of urea were selected to record the change of chemical shift, and 1:1 (ligand: ion) fitting was performed with BindFit software.

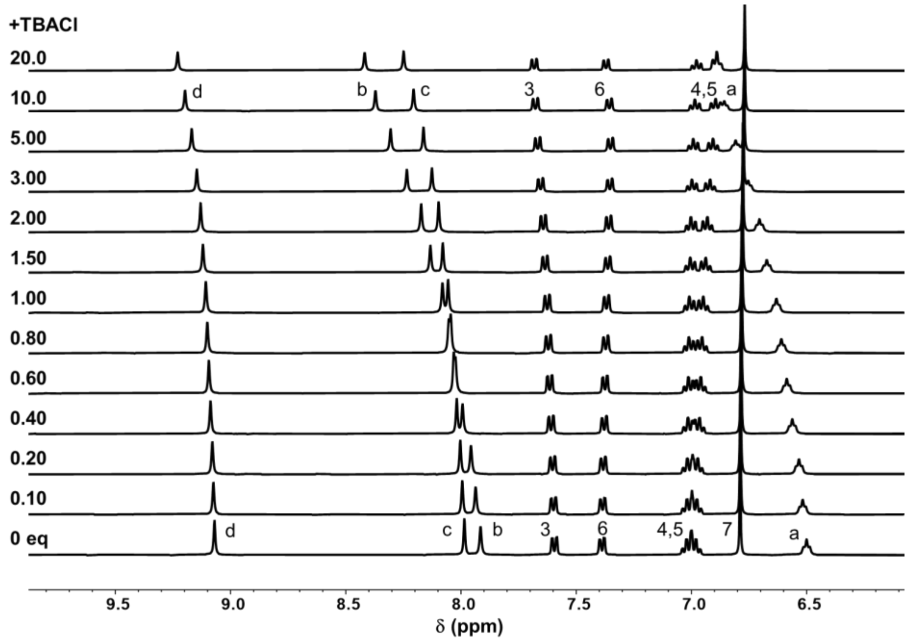

Figure S16. Stacked partial ^{1}H NMR spectra (400 MHz, 298 K, DMSO-d_6) of receptor $\mathbf{L}^1$ by adding chloride as tetrabutylammonium salt (TBACl). ([$\mathbf{L}^1$] = 2 mM, [TBACl] = 50 mM)

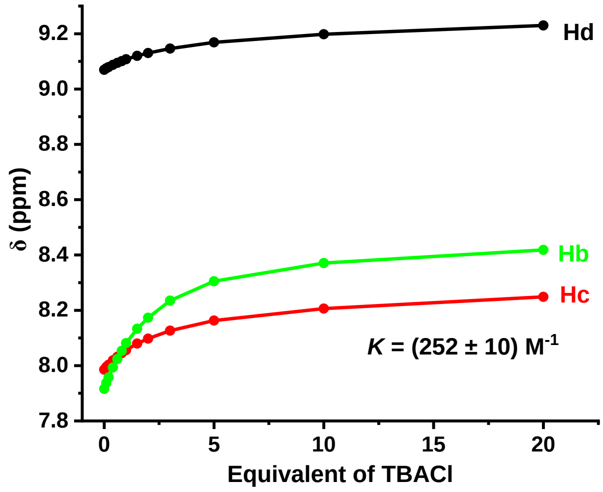

Figure S17. Chemical shift changes of proton H_d, H_c and H_b during titration. The chloride binding affinity was determined to be (215 ± 7) M^{-1} using Bindfit (v0.5).

The results in Figure S18 indicated that HCO_3^- and the ligand were quickly exchanged. The hydrogen peaks of urea were selected to record the change of chemical shift, and 1:1 (ligand: ion) fitting was performed with BindFit (v0.5).

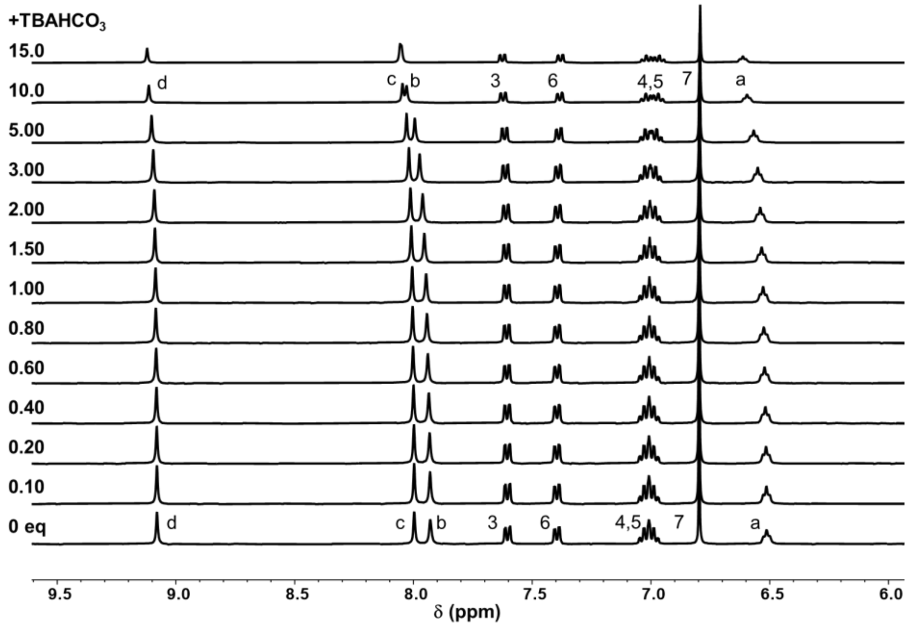

Figure S18. Stacked partial ^{1}H NMR spectra (400 MHz, 298 K, DMSO-d_6) of receptor $\mathbf{L}^1$ by adding chloride as tetrabutylammonium salt (TBAHCO$_3$). ([$\mathbf{L}^1$] = 2 mM, [TBAHCO$_3$] = 50 mM)

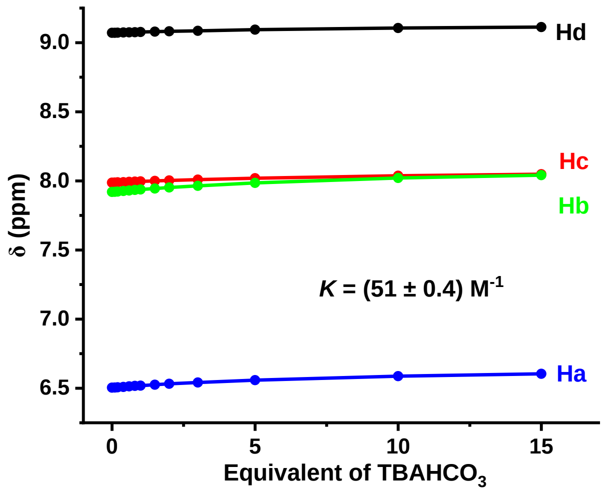

Figure S19. Chemical shift changes of proton H_d, H_c, H_b and Ha during titration. The chloride binding affinity was determined to be (51 ± 0.4) M^{-1} using BindFit (v0.5).

TBA$_2$HPO$_4$ is added directly to the ligand solution. The results in Figure S20 indicated that HPO_4^{2-} and the ligand were slowly exchanged. The characteristic peaks with obvious peak position changes and accurate integration were selected for integration, and the corresponding concentration of the complex was calculated according to the integral area. The obtained data were fitted and analyzed using Origin to obtain the binding constants of the HPO_4^{2-} and the ligand.

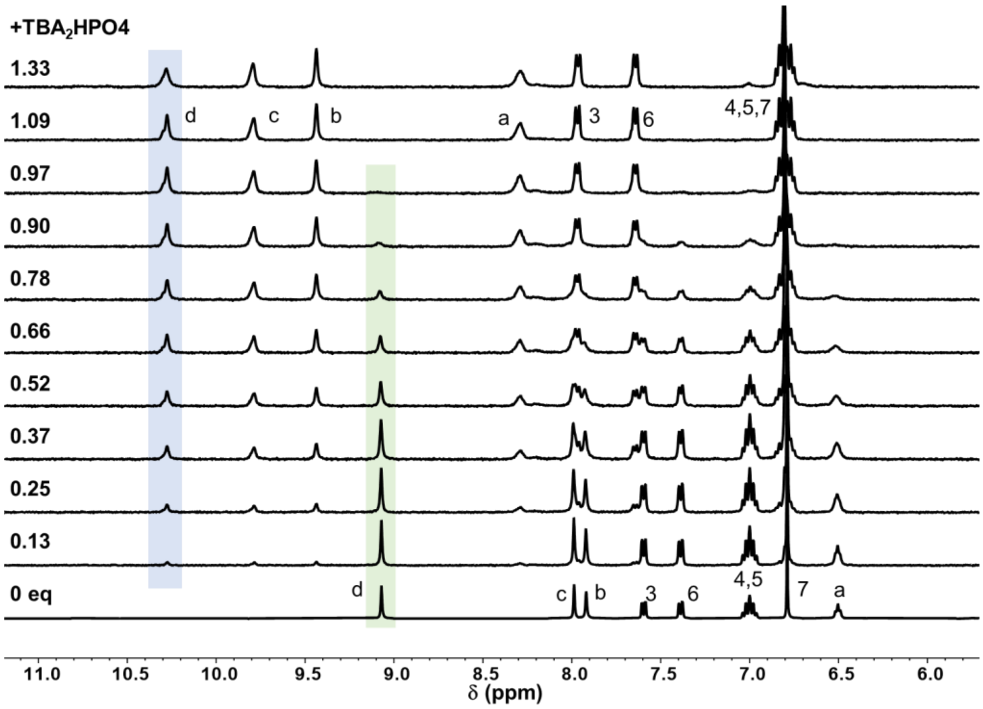

Figure S20. Stacked partial ^{1}H NMR spectra (400 MHz, 298 K, DMSO-d_6) of receptor **L**[1] by adding TBA_2HPO_4. ([**L**[1]] = 2 mM, [TBA_2HPO_4] = 50 mM)

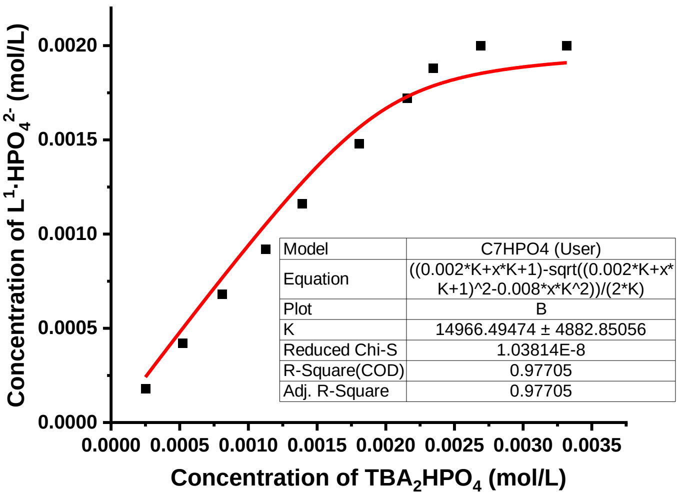

Figure S21. Fitted curve for the ^{1}H NMR titration between **L**[1] and HPO_4^{2-}, which is derived from Figure S20. It indicates that the binding constant of the ligand to HPO_4^{2-} is larger than 10^4 M^{-1}.

By adding $H_2PO_4^-$directly to the ligand solution, the dihydrogen phosphate hydrolyzed to HPO_4^-, so the binding constant could not be determined. The result is shown in the Figure S22.

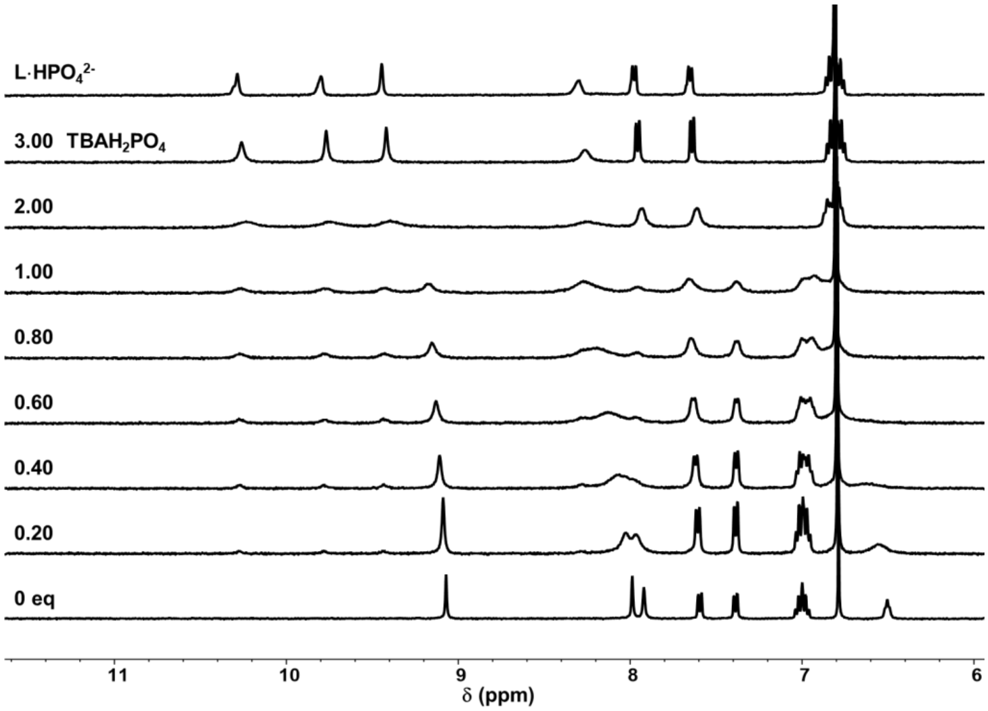

Figure S22. Stacked partial ^{1}H NMR spectra (400 MHz, 298 K, DMSO-d_6) of receptor $\mathbf{L}^1$ by adding $TBAH_2PO_4$. ([$\mathbf{L^1}$] = 2 mM, [$TBAH_2PO_4$] = 50 mM)

Due to the phenomenon of peak broadening when titrating sulfate directly into ligand, it is difficult to measure the binding constant. Therefore, competitive titration of sulfate and chloride ions is used to measure the binding constant between ligand and sulfate. The results in Figure S23 indicated that SO_4^{2-} and the L·Cl⁻ were slowly exchanged. The characteristic peaks with obvious peak position changes and accurate integration were selected for integration, and the corresponding concentration of the complex was calculated according to the integral area. The obtained data were fitted and analyzed using Origin to obtain the competitive exchange constants between sulfate and chloride.

The detailed calculation steps are as follows:

Competing titration equation： $L^1 \cdot Cl^- + SO_4^{2-} \rightleftharpoons L^1 \cdot SO_4^{2-} + Cl^-$

For the calculation, we defined these:

the total concentration of TBA_2SO_4 added is **x**， $[L^1 \cdot SO_4^{2-}]$ = **y**，

the initial concentration of $L^1 \cdot Cl^-$ is **b**，

thus $[SO_4^{2-}] = x - y$， $[Cl^-] = y$，$[L^1 \cdot Cl^-] = b - y$

$$K = \frac{[L^1 \cdot SO_4^{2-}]*[Cl^-]}{[L^1 \cdot Cl^-]*[SO_4^{2-}]} = \frac{y^2}{(b-y)(x-y)} = \frac{y^2}{y^2-(x+b)y+bx}$$

$$\frac{1}{K} = \frac{y^2-(x+b)y+bx}{y^2}$$

$$(\frac{1}{K} - 1)y^2 + (x+b)y - bx = 0$$

$$y = \frac{-(x+b) \pm \sqrt{(x+b)^2 + 4bx(\frac{1}{K} - 1)}}{2(\frac{1}{K} - 1)}$$

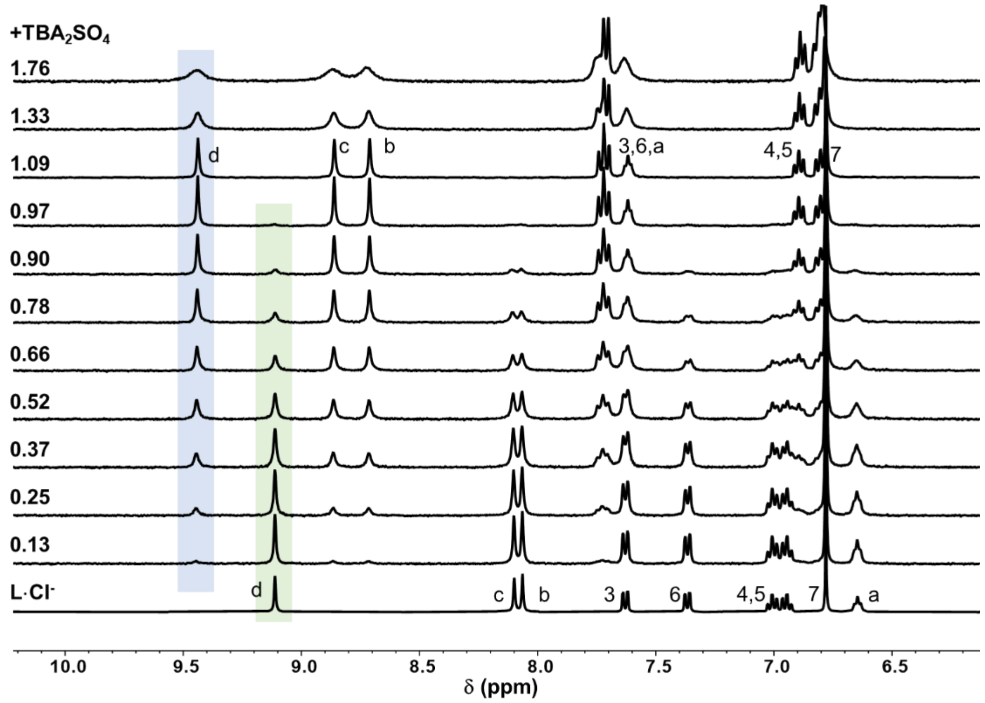

Figure S23. Stacked partial ^{1}H NMR spectra (400 MHz, 298 K, DMSO-d_6, 2 mM) of chloride binding complex by adding TBA_2SO_4 (50 mM).

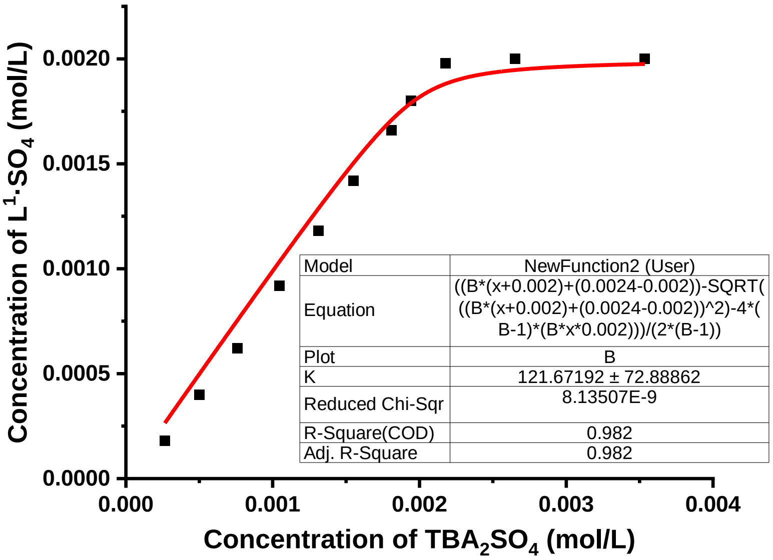

Figure S24. Fitted curve for the competitive titration between sulfate and chloride anions, which is derived from Figure S23. It indicates that sulfate binding affinity is 121-fold stronger than chloride. It can be concluded that the binding constant of ligand and sulfate is larger than 10^4 M^{-1}.

The ligand-anion binding constants are summarized in Table S4.

Table S4 Approximate binding constants (M^{-1}) of L^1 with anions in DMSO-d_6

Anion	SO_4^{2-}	HPO_4^{2-}	$H_2PO_4^-$	Cl^-	HCO_3^-
K	$(3.4 \pm 2) \times 10^4$	$(1.5 \pm 0.5) \times 10^4$	*	(252 ± 10)	(51 ± 0.4)

* The results can not be fitted.

This suggests that the binding constants of L^1 to common anions are much smaller than that of L^1 to CA4P, as further demonstrated by the competitive titration in DMSO-d_6. We selected the anion (SO_4^{2-}) with the highest binding ability for the competitive study. As shown in Figure S25, upon addition of 1 equiv. of CA4P to the $L^1 \cdot SO_4^{2-}$ complex, the ^{1}H NMR spectra of complex $L^1 \cdot SO_4^{2-}$ disappeared and a new set of peaks corresponding to the L^1·CA4P complex appeared, indicating that L^1 prefers binding to anionic CA4P compared to other common anions.

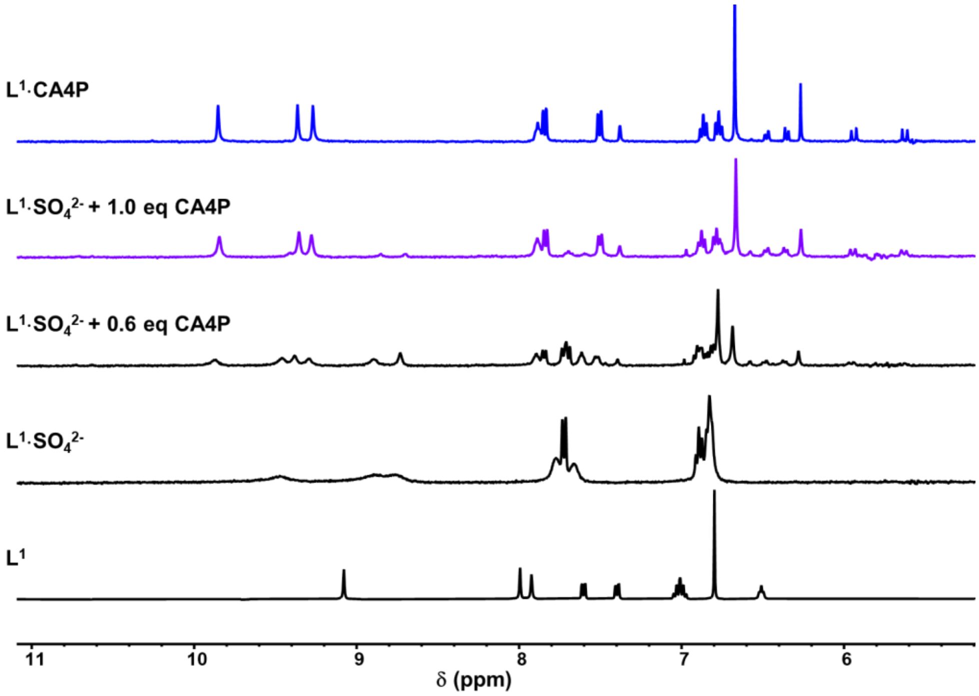

Figure S25. Stacked partial ^{1}H NMR spectra (400 MHz, 298 K, DMSO-d_6, 2 mM) of sulfate binding complex by adding CA4P (50 mM).

<u>S9. Cell experimental conditions</u>

Cell culture: HeLa cells were incubated in complete medium (Dulbecco's modified Eagle's Medium (DMEM), supplemented with 10% fetal bovine serum (FBS) and 1% penicillin-streptomycin) at 37°C in atmosphere containing 5% CO_2.

Preparation of cell culture medium: Cell culture medium provides cell growth and cell proliferation with a multi-nutrient rich liquid consisting of 90% DMEM (base culture medium), 10% FBS (fetal bovine serum), and 1% (volume fraction) penicillin-streptomycin solution (double antibody). These materials were stored in a 4°C refrigerator and preheated to 37°C before use.

Preparation of PBS: Weigh 8 g NaCl, 0.2 g KCl, 3.63 g $Na_2HPO_4 \cdot 12H_2O$, 0.24 g KH_2PO_4 into 1000 mL wide-mouth bottle and add 1000 mL deionized water. Adjust pH to 7.2-7.4 with 0.1 M HCl or NaOH. PBS should be sterilized with autoclave before use.

HeLa cells were seeded in flat-bottomed 96-well plates, 1×10^4 cells per well, with 200 μL complete culture media for 24 h. After suck out the old medium, the HeLa cells were incubated with different concentrations of complex. All stock solutions were prepared in DMSO (5 mM) and diluted with complete medium. After cultured for 48 h, the cells were washed with PBS (pH 7.4) two times. 10 μL Cell Counting Kit-8 (CCK-8) solution and 90 μL DMEM were added per well simultaneously. After 40 minutes, the absorbance at 450 nm was read by 96-well plates reader.

The viability of Hela cells was calculated by the following equation:

$$CV = (As\text{-}Ab) / (Ac\text{-}Ab) \times 100\%$$

CV stands for the viability of cells, As, Ac and Ab stand for the absorbance of cells containing complex cell control (0 μM complex) and blank control (wells containing neither cells nor complex).
